# Global, regional, and national burden and trends of reproductive-aged male and female infertility from 1990–2021

**DOI:** 10.3389/fendo.2025.1506229

**Published:** 2025-09-04

**Authors:** Guoqiang Zeng, Lingyun Liu, Yuantao Wang, Jinyu Yu, Hongliang Wang, Faping Li

**Affiliations:** ^1^ Department of Andrology, The First Hospital of Jilin University, Changchun, Jilin, China; ^2^ Department of Urology II, The First Hospital of Jilin University, Changchun, Jilin, China

**Keywords:** global burden of disease study, infertility, reproductive age, prevalence, disability-adjusted life-years

## Abstract

**Background:**

Infertility is a complex condition influenced by multiple factors and is associated with significant health and social implications. The aim of this study was to investigate the burden of infertility among reproductive-aged (15–49 years) men and women from 1990 to 2021, along with the associated trends.

**Methods:**

Data on the prevalence and disability-adjusted life years (DALYs) related to infertility among 15–49 years men and women from 1990 to 2021 were obtained from the Global Burden of Disease (GBD) Study 2021. The estimated annual percentage change (EAPC) was calculated to assess the changes in age-standardized rates of prevalence (ASPR) and DALYs (ASDR). Additionally, the relationship between disease burden and the socio-demographic Index (SDI) was analyzed. Joinpoint regression analysis was employed to conduct a thorough examination of the trends for disease burden from 1990 to 2021.

**Results:**

In 2021, the global number of cases and DALYs for male infertility among 15–49 years increased by 74.66% and 74.64% since 1990. For females, the global number of cases and DALYs increased by 84.44% and 84.43% respectively. Among the five SDI regions, the middle SDI region recorded the highest number of cases and DALYs in 2021, accounting for approximately one-third of the global total. From an age subgroup perspective, the 35–39 age group reported the highest number of cases in 2021. The infertility disease burden was negatively correlated with SDI in national level. Joinpoint analysis demonstrated a declining trend in the annual percentage change (APC) for male infertility ASPR and ASDR during the periods 1990–2001 and 2005-2010. The ASPR time points of female infertility were slightly different from those for males, but the overall trend remained consistent.

**Conclusion:**

Overall, the burden of infertility among 15–49 years men and women has significantly increased globally over the past 32 years, particularly in middle SDI regions and among the 35–39 age group. The findings underscore the importance of tailored interventions aimed at addressing infertility issues in this population, contributing to the achievement of the sustainable development goals established by the World Health Organization.

## Introduction

Clinical infertility is defined as the inability of a couple to conceive after one year of regular and unprotected intercourse (1). This condition can lead to a range of challenges, including psychological distress, financial strain, social stigma, and marital disharmony (2–4). While medical treatments and assisted reproductive technologies have improved pregnancy outcomes, their application is often limited by ethical considerations, economics, strict delivery conditions, and post-delivery health statuses (5). As the global population continued to grow and society rapidly evolves, an increasing number of individuals faced the direct threat of infertility. This issue currently affected at least 180 million couples of reproductive age worldwide, posing challenges for countless families and society, while also placing a heavier burden on healthcare systems (6). Consequently, infertility has emerged as a significant public health issue and is included in the World Health Organization’s global burden of disease (GBD) assessments.

The causes of infertility are diverse. Male infertility may stem from issues such as sperm dysplasia and sexual dysfunction, while female infertility can arise from conditions like pelvic damage and ovulation disorders (5). Additionally, emotional stress plays a significant role in contributing to infertility (7). According to recent studies, approximately 8.8% of women of reproductive age in the United States experienced infertility (8). In China, the situation was even more concerning, with over 40 million individuals affected by infertility—a number that increased by several hundred thousand each year—resulting in a prevalence rate of 12.5% to 15% among the population of reproductive age (9). Overall, infertility tends to be more prevalent in women than in men. It was estimated that 20% to 30% of infertility cases could be attributed to male factors (6). In the United Kingdom, the estimated prevalence of infertility stood at 12.5% for women and 10.1% for men (10). Notably, the prevalence of infertility varied significantly across regions, ethnicities, and cultural contexts (11). In economically developed countries, rates ranged from 3.5% to 16.7%, while in low-income countries, they varied from 6.9% to 9.3% (12). Given these variations, it is imperative for policymakers and researchers to have access to comprehensive, up-to-date epidemiological data to formulate effective strategies to address the needs of reproductive-age populations. Despite several studies documenting the burden of infertility, they are not comprehensive, highlighting the need for further research in this area (13, 14).

The GBD study serves as a robust framework for elucidating the prevalence, distribution, and trends of infertility, employing a diverse array of statistical methodologies to derive population health indicators from global-level data. In this investigation, we utilized the GBD statistical modeling system to assess the disease burden of infertility among individuals of reproductive age (15–49 years) over the period from 1990 to 2021. This included a comprehensive analysis of prevalence and disability-adjusted life years (DALYs). Furthermore, we examined the relationship between the socio-demographic index (SDI) and the level of development, alongside demographic trends in the disease burden over time. The insights gained from this analysis are intended to assist clinicians, epidemiologists, and health policymakers in optimizing the allocation of healthcare resources and in formulating more effective public health strategies.

## Method

### Data source

The GBD is a global research initiative led by the Institute for Health Metrics and Evaluation (IHME) in collaboration with various international health organizations. The project aims to comprehensively assess the impact of diseases, injuries, and risk factors on human health. The GBD study involves gathering data from various sources, and following the data collection process, potential biases within each dataset are evaluated and adjusted using the Bayesian meta-regression tool DisMod-MR 2.1 for standardized statistical modeling. The database is accessible for search and download via the official GBD website (https://www.healthdata.org/research-analysis/gbd). The GBD 2021 offers comprehensive data on prevalence, incidence, mortality, DALYs, and age-standardized rate (ASR) associated with 369 health hazards spanning various diseases and injuries, alongside 88 risk factors across 204 countries and territories (15). The Ethics Committee of the First Hospital of Jilin University determined that no approval was needed in this study because all data used were publicly available with no disclosure of personal information or privacy involved.

### Burden description

In this study, we analyzed data pertaining to the prevalence of reproductive-aged male and female infertility, alongside their respective DALYs estimates and 95% uncertainty intervals (UI) from 1990 to 2021. According to the World Health Organization, the reproductive age range is defined as 15 to 49 years (16). Consequently, the data analyzed in this study were categorized into seven age subgroups: 15–19 years, 20–24 years, 25–29 years, 30–34 years, 35–39 years, 40–44 years, and 45–49 years.

DALYs are an indicator used to quantify the burden of disease. It represents the total number of years of healthy life lost due to disease or premature death and is the sum of YLL (Years of Life Lost) and YLD (Years Lived with Disability). YLD are calculated by multiplying the number of affected individuals by the duration of their remission or the time until death, adjusted for the severity of their disability. Conversely, YLL are computed by multiplying the total number of deaths by the corresponding standard life expectancy derived from the reference life table.

### Socio-demographic index

Furthermore, this study employed the SDI, a composite indicator designed to assess the development levels of regions or countries. The SDI calculation incorporates three key components: the total fertility rate among individuals under the age of 25, the average education level of those aged 15 and older, and the lagging distribution index of per capita income. The SDI value ranges from 0 to 1, with higher values indicating a greater socio-economic status. Based on the SDI values for 2021, 204 countries and territories have been categorized into five distinct groups: high SDI (≥0.80), middle-high SDI (≥0.69 and <0.80), middle SDI (≥0.61 and <0.69), middle-low SDI (≥0.45 and <0.61), and low SDI (<0.45) (17).

### Age-standardized rate

The ASR is designed to mitigate the influence of age distribution within the population, thereby enhancing the comparability of research indicators. It represents a weighted average of the age-specific ratios per 100,000 individuals, with weights derived from the standard population’s age distribution. This study calculated the ASR for individuals aged 15 to 49 years. Specifically, we focused on the age-standardized prevalence rate (ASPR) and the age-standardized annual disability-adjusted life years rate (ASDR). These indicators were derived from the GBD database using world population age criteria and were computed according to the following formula: $ASR=\frac{\sum_{i=1}^{N}\alpha_{i}W_{i}}{\sum_{i=1}^{N}W_{i}}\times 100,000$ . In this equation, represents the age-specific ratio for the fourth age group, while denotes the number of individuals within the same age group based on the GBD 2021 standard population. Here, *N* refers to the total number of age groups (18).

### Estimated average percentage change

To examine trends of ASPR and ASDR, we calculated the estimated average percentage change (EAPC), a widely used metric for trend quantification. The EAPC was derived by modeling the relationship between the n aural logarithm of ASR and time using the equation: ln (ASR)=α+βx+ε . The EAPC and its corresponding 95% confidence interval (CI) were computed using the formula: $EAPC_{\text{with}95\%CI}=100\times \left(e^{\beta }-1\right)$ . A 95% CI that has an upper limit less than 0 indicates a declining ASR over time, whereas a lower limit greater than 0 suggests an increasing ASR. In cases where the 95% CI includes 0, we interpret the change in ASR as statistically insignificant (18).

### Joinpoint regression analysis

In this study, we employed the Joinpoint regression analysis model, a statistical technique commonly utilized in epidemiological research to evaluate trends in disease prevalence and mortality (19). This modeling approach can effectively identify and quantitatively describe significant points of change within time series data related to infertility prevalence at global, regional, and national levels. The model facilitates the calculation of the annual percentage change (APC) alongside its corresponding 95% CI, providing insight into the prevalence trends over the specified timeframe. To offer a comprehensive assessment of the observed trends, we also calculated the average annual percentage change (AAPC), which includes general trend data for the study period from 1990 to 2021. From a statistical perspective, an APC or AAPC estimate that includes a 95% CI lower limit greater than 0 suggests an upward trend within the specified interval. Conversely, if the upper limit of the 95% CI is below 0, this indicates a downward trajectory. When the 95% CI of the APC or AAPC encompasses 0, it implies that the trend is stable (19).

All statistical analyses and graphical representations were conducted using *R* software (version 4.2.2) and GraphPad Prism.

## Results

### Global level

**Figure 1:**
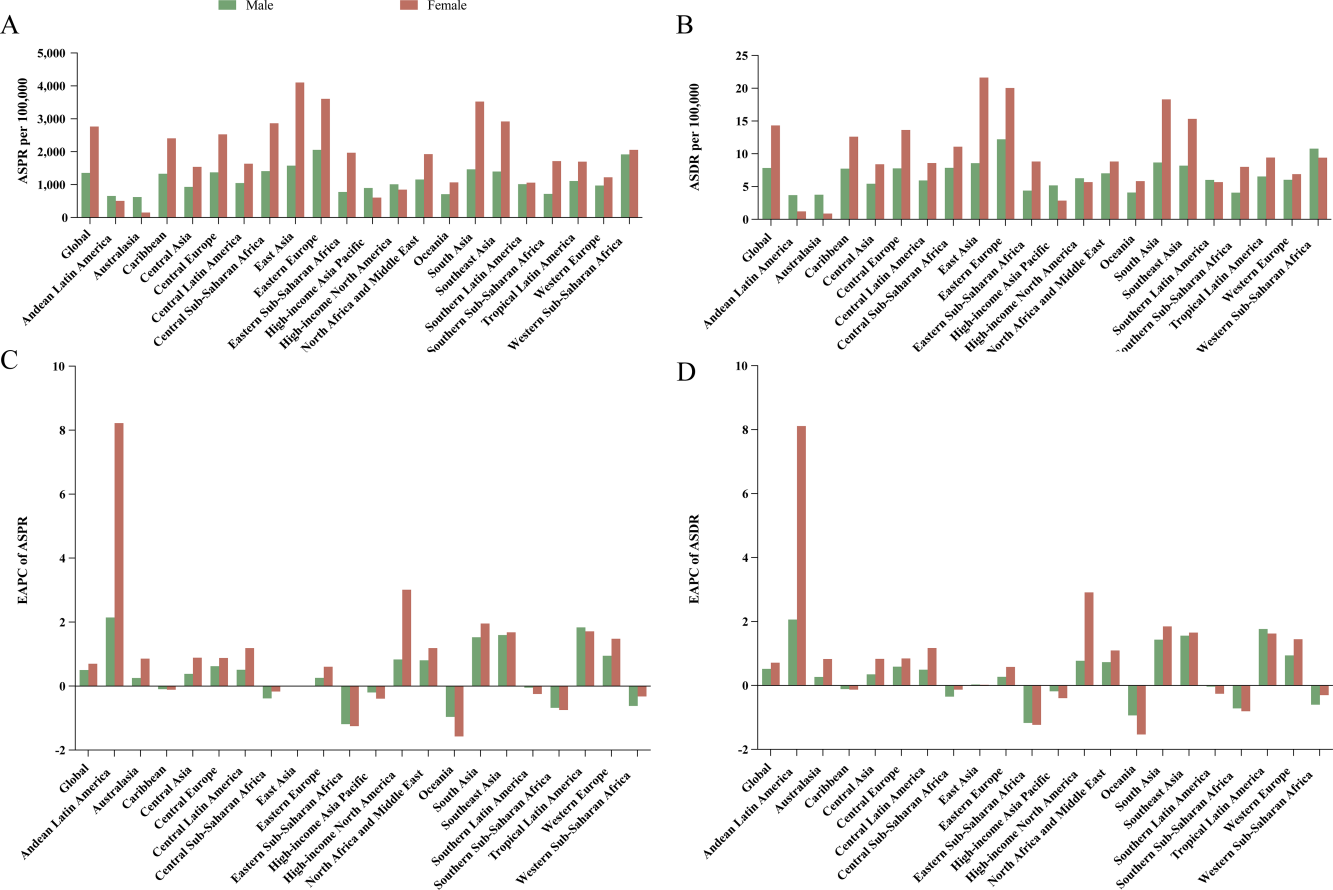
Age-standardized prevalence and DALYs rates in 2021, and their estimated annual percentage changes from 1990 to 2021 for male and female infertility in global and 21 regions. Age-standardized rates of prevalence **(A)** and disability-adjusted life-years **(B)**, and estimated annual percentage changes of age-standardized rates of prevalence **(C)** and disability-adjusted life-years **(D)**. ASPR, age-standardized prevalence rate; ASDR, age-standardized disability-adjusted life-year rate; EAPC, estimated annual percentage change.

In 2021, the total cases of reproductive-aged male infertility globally were approximately 55,000,818 (95% UI: 32,611,257 - 88,727,953), representing a substantial increase of 74.66% compared to 1990. The ASPR for men in 2021 was 1,354.76 per 100,000 (95% UI: 802.12 - 2,174.77), reflecting a rise of 16.90% since 1990. Moreover, the EAPC was 0.50, indicating an increase of 0.50 cases per 100,000 individuals from 1990 to 2021 (95% UI: 0.36 - 0.64). In 2021, the total cases of reproductive-aged female infertility worldwide among were approximately 110,089,459 (95% UI: 58,608,815 - 195,025,585), marking an increase of 84.44% relative to 1990. The ASPR for women in 2021 was 2,764.62 per 100,000 (95% UI: 1,476.33 - 4,862.57), which was a 21.94% rise since 1990. The EAPC for women was 0.70 (95% UI: 0.53 - 0.87). In 2021, global DALYs for reproductive-aged men amounted to approximately 317,614 (95% UI: 116,288 - 752,758), while for women, it was around 601,134 (95% UI: 213,158 - 1,468,475). The increase in DALYs for women (84.43%) was greater than that for men (74.64%) since 1990. From 1990 to 2021, the ASDR for both genders exhibited an upward trend. Notably, the EAPC for women [0.71 (95% UI: 0.54 - 0.88)] was higher than that for men [0.51 (95% UI: 0.38 - 0.65)] (**Table 1** and **Figure 1**). Overall, the burden of infertility was greater in women than in men.

**Table 1:** Global prevalence and DALYs of infertility from 1990 to 2021.

Year	Male	Female
1990		
Prevalence (95% UI)	31,490,382 (18,725,068 - 50,165,061)	59,690,000 (32,625,584 - 104,614,493)
DALYs (95% UI)	181,869 (66,532 - 425,579)	325,937 (114,823 - 807,747)
ASPR/100,000 (95% UI)	1,158.86 (696.62 - 1,858.35)	2,267.26 (1,219.63 - 3,969.94)
ASDR/100,000 (95% UI)	6.65 (2.47 -15.61)	12.32 (4.39 - 30.93)
2021		
Prevalence (95% UI)	55,000,818 (32,611,257 - 88,727,953)	110,089,459 (58,608,815 - 195,025,585)
DALYs (95% UI)	317,614 (116,288 - 752,758)	601,134 (213,158 - 1,468,475)
ASPR/100,000 (95% UI)	1,354.76 (802.12 - 2,174.77)	2,764.62 (1,476.33 - 4,862.57)
ASDR/100,000 (95% UI)	7.84 (2.85 - 18.56)	15.12 (5.35 - 36.88)
1990-2021		
Prevalence (%)	74.66	84.44
DALYs (%)	74.64	84.43
EAPC of ASPR (95% CI)	0.50 (0.36 - 0.64)	0.70 (0.53 - 0.87)
EAPC of ASDR (95% CI)	0.51 (0.38 - 0.65)	0.71 (0.54 - 0.88)

DALYs, disability-adjusted life-years; ASPR, age-standardized prevalence rate; ASDR, age-standardized disability-adjusted life-years rate; EAPC, estimated annual percentage change; UI, uncertainty interval; CI, confidence interval.

### Regional level

In 2021, the five regions with the highest prevalence and DALYs of infertility among both reproductive-aged males and females were South Asia, East Asia, Southeast Asia, North Africa and Middle East, and Western Sub-Saharan Africa (**Supplementary Table S1**). Among men, Western Sub-Saharan Africa exhibited the highest ASPR at 2,058.13 per 100,000 (95% UI: 1,120.20 - 3,444.23), whereas East Asia reported the highest ASPR for women at 4,102.68 per 100,000 (95% UI: 2,124.47 - 7,170.94). Notably, regions such as Andean Latin America, Australasia, High-income Asia Pacific, and High-income North America recorded higher cases and ASPRs for reproductive-aged male infertility than for female infertility (**Figure 1A** and **Supplementary Table S2**). The ASDR for male infertility was highest in Eastern Europe at 12.20 per 100,000 (95% UI: 4.27 - 30.22), while female infertility had the highest ASDR in East Asia at 21.55 per 100,000 (95% UI: 7.40 - 54.10) (**Figure 1B** and **Supplementary Table S2**). Most regions demonstrated an upward trend in ASPR for infertility (EAPC > 0). Regions with an EAPC < 0 included all areas within Sub-Saharan Africa. It is particularly noteworthy that from 1990 to 2021, both male and female ASPRs in Andean Latin America increased significantly, with EAPC values of 2.14 (95% UI: 1.77 - 2.51) and 8.22 (95% UI: 6.70 - 9.76), respectively. Conversely, there was a notable decline in male infertility in Eastern Sub-Saharan Africa, with an EAPC of -1.19 (95% UI: -1.41 to - 0.96), and a marked decrease in female infertility in Oceania, where the EAPC was -1.58 (95% UI: -1.85 to - 1.30) (**Figure 1C** and **Supplementary Table S3**). It is worth noting that, in most regions, changes in ASPR and ASDR for women were more pronounced than those for men, and the trends in ASDR—whether increasing or decreasing—were consistent with those observed in ASPR (**Figures 1C** and **D**, **Supplementary Table S3**).

### National level

**Figure 2:**
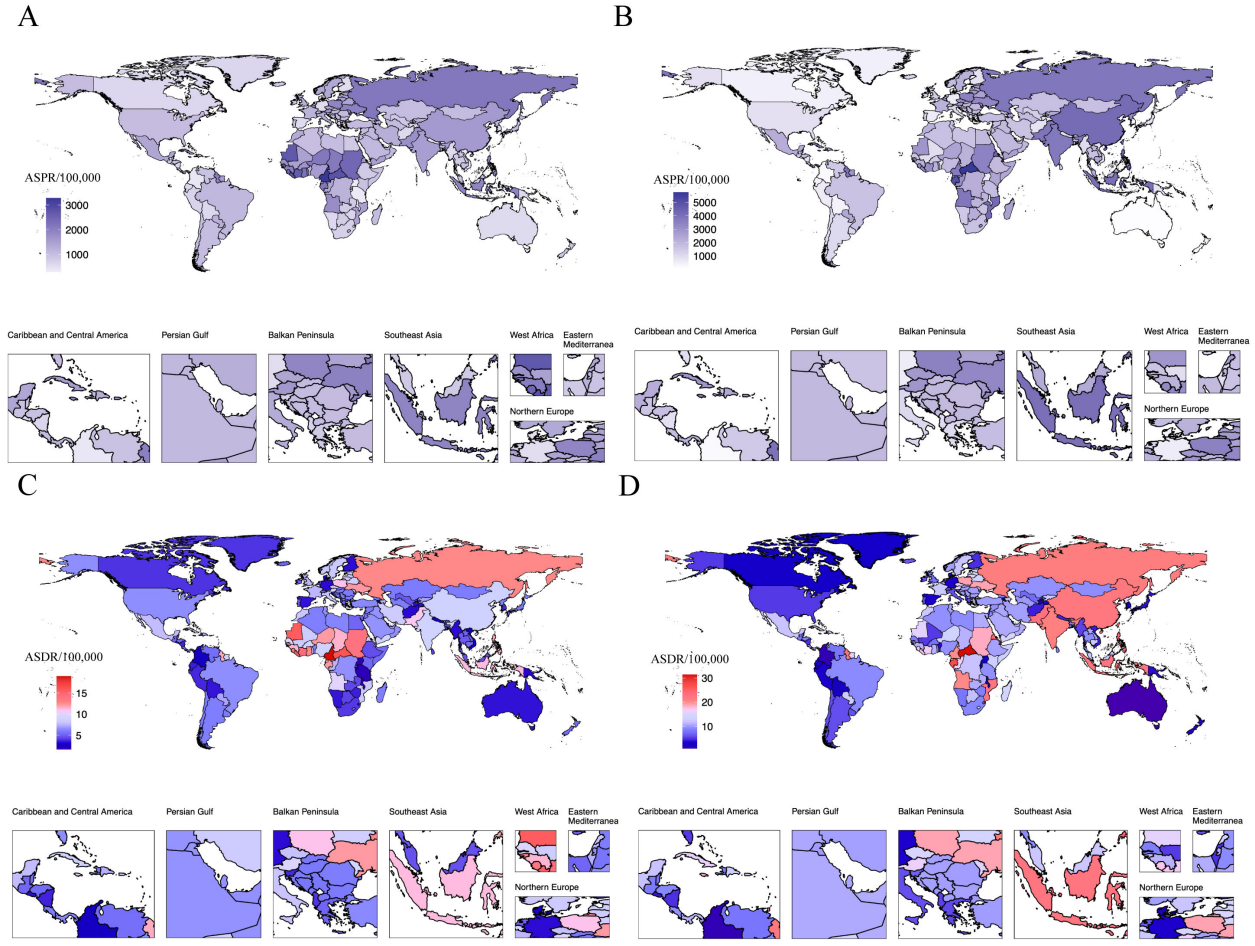
National age-standardized prevalence and disability-adjusted life-years rates in 2021. Age-standardized rates of prevalence for male infertility **(A)** and 582 female infertility **(B)**; Age-standardized rates of disability-adjusted life-years for male infertility **(C)** and female infertility **(D)**. ASPR, age-standardized prevalence rate; ASDR, age-standardized disability-adjusted life-year rate.

In 2021, the three countries with the highest number of reproductive-aged male infertility cases were India [12,352,775 (95% UI: 7,079,212 - 20,281,847)], China [11,845,804; 95% UI: 6,488,726 - 20,756,171)], and Indonesia [3,096,051 (95% UI: 1,794,084 - 5,069,623)]. For female infertility, the top three countries were China [29,317,000 (95% UI: 14,569,167 - 52,098,692)], India [29,075,289 (95% UI: 16,070,794 - 49,483,699)], and Indonesia [6,251,542 (95% UI: 3,293,414 - 11,133,561)], same as male infertility (**Supplementary Table S4**). The country with the highest ASPR for male infertility was Cameroon, with 3,280.58 per 100,000 (95% UI: 1,939.56 - 5,141.30), while for female infertility, China reported the highest ASPR at 4,144.55 per 100,000 (95% UI: 2,149.99 - 7,228.30) (**Figures 2A, B**, **Supplementary Table S5**). From 1990 to 2021, the country with the highest increase in ASPR for males was the Philippines [5.33 (95% UI: 3.27 - 7.44)], whereas for females, Ecuador saw the most significant rise [9.33 (95% UI: 7.27 - 11.42)]. The most notable decrease in ASPR for both male and female infertility was observed in Malawi, with EAPC of -4.22 (95% UI: -4.55 to -3.90) and -6.2 (95% UI: -6.65 to -5.75), respectively. Among the 204 countries analyzed, 96 showed an EAPC > 0 for male infertility, while 104 countries exhibited the same for female infertility (**Supplementary Table S6**, **Supplementary Figures S1A, B**). For DALYs related to reproductive-aged male infertility, the top three countries were India [72,582 (95% UI: 26,125 - 167,575)], China [63,931 (95% UI: 21,752 - 155,614)], and Indonesia [17,850 (95% UI: 6,496 - 43,630)]. In terms of female infertility, the countries with the highest DALYs were India [161,474 (95% UI: 57,797 - 392,596)), China [153,252 (95% UI: 50,580 - 396,547)], and Indonesia [34,094 (95% UI: 11,773 - 82,100)] (**Supplementary Table S4**). The highest ASDR for males was found in Cameroon [18.96/100,000 (95% UI: 0.04 - 44.69)], while for females, the Central African Republic reported the highest ASDR at 31.40 per 100,000 (95% UI: 11.58 - 71.65) (**Figures 2C, D**, **Supplementary Table S5**). The most significant increase in male ASDR was observed in the Philippines [5.28 (95% UI: 3.29 - 7.30)], and for females, Ecuador demonstrated a notable rise [9.15 (95% UI: 7.13 - 11.20)]. Both males and females experienced the most pronounced declines in ASDR in Malawi, with EAPC values of -4.31 (95% UI: -4.64 to -3.99) and -6.18 (95% UI: -6.62 to -5.73), respectively (**Supplementary Table S6**, **Supplementary Figures S1C, D**).

### Age patterns

**Figure 3:**
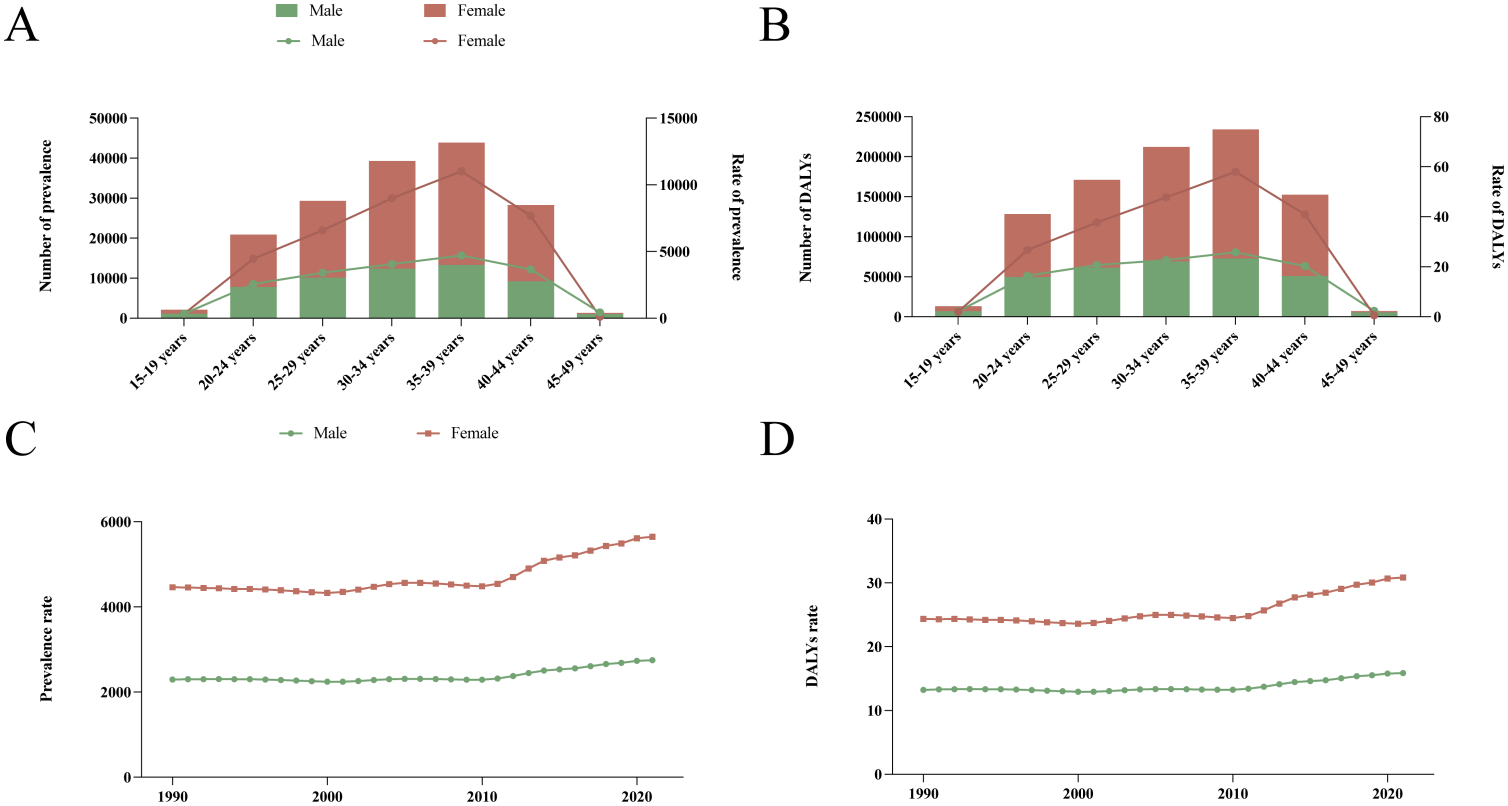
The cross-sectional (2021) and longitudinal trends (1990–2021) of prevalence rate and DALYs rate of reproductive-aged male and female infertility. Number and rate of prevalence **(A)** and DALYs **(B)** in 2021; prevalence rate **(C)** and DALYs rate **(D)** in 1990-2021. DALYs, disability-adjusted life-years.

In 2021, the prevalence of infertility and associated DALYs for both males and females were predominantly observed in the age subgroup of 35 to 39 years, exhibiting a pyramid-like age distribution (**Figures 3A, B**). In the 15–19 and 45–49 age subgroups, the prevalence and DALYs rates for male infertility exceeded those for females; however, in all other age groups, the prevalence rates for females were higher than those for males (**Figures 3A, B**). Notably, within the five age subgroups of 20 to 44 years, there were substantial differences in ASPR and ASDR between genders. From 1990 to 2021, both the prevalence and DALY rates for infertility among individuals aged 15–49 showed an upward trend. Throughout this period, the rates for females remained consistently higher than those for males (**Figures 3C, D**). Specifically, from 1990 to 2021, there was a general increase in the disease burden for the 15–19 age subgroup, although trends varied between genders: females showed a declining trend from 1995 to 2010, while males exhibited a decrease from 1995 to 2000, followed by a gradual increase thereafter (**Supplementary Figures S2A, S3A**). In the remaining age subgroups, the disease burden experienced slight fluctuations, but the overall trend indicated an increase (**Supplementary Figures S2B–G, S3B–G**). Consequently, the burden of infertility primarily concentrated within the 35–39 age subgroup.

### The association between infertility burden and SDI

**Figure 4:**
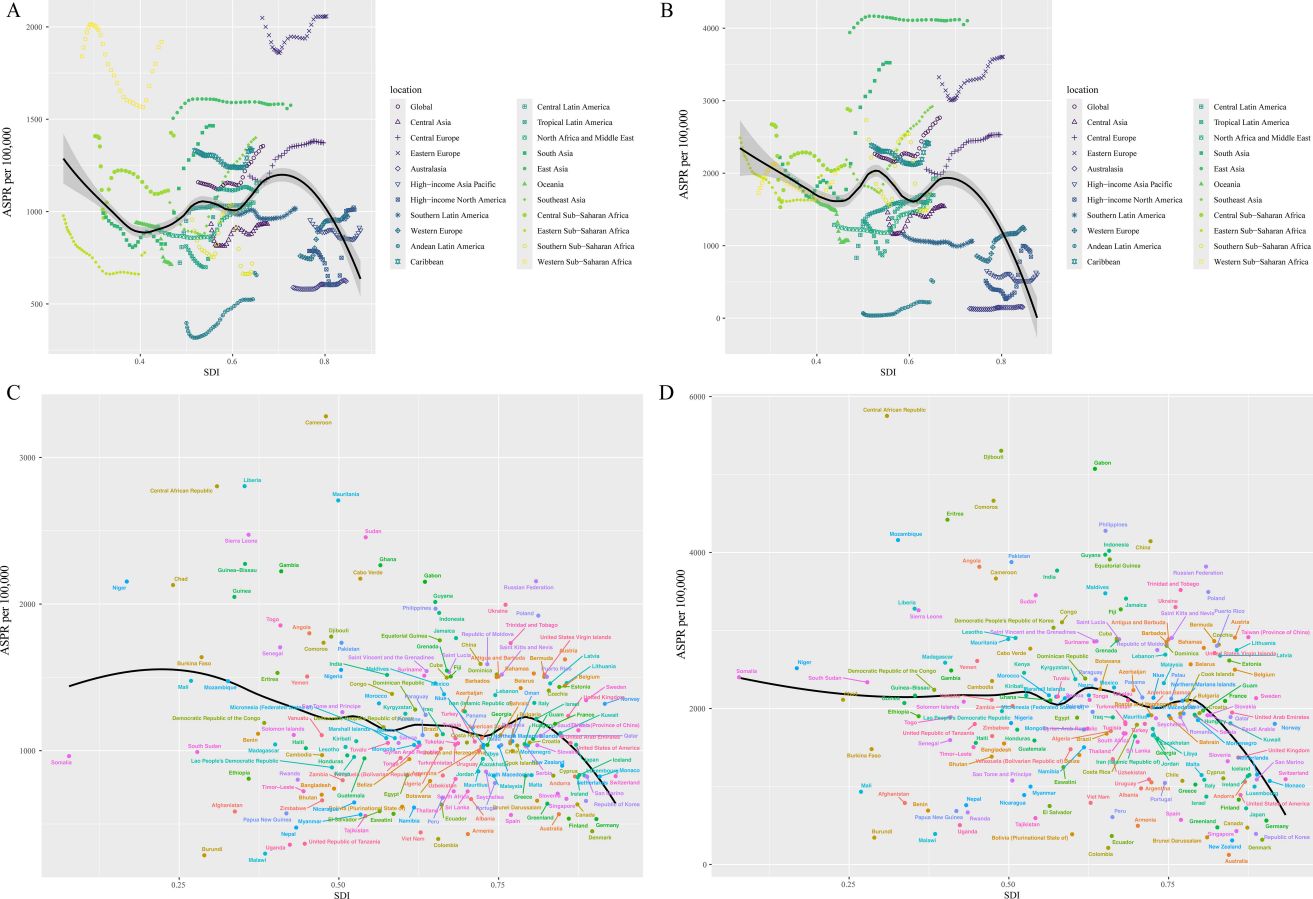
ASPRs for male and female fertility of 21 regions and 204 countries and territories by SDI. ASPRs for male **(A)** and female **(B)** fertility of 21 regions from 1990−2021 by SDI; ASPRs for male **(C)** and female **(D)** fertility of 204 countries and territories in 2021 by SDI. ASPR, age-standardized prevalence rate, SDI, socio-demographic index.

In 2021, the highest cases and DALYs for reproductive-aged male infertility were observed in middle SDI regions, accounting for approximately one-third of the global total. The estimated number of affected males was 18,151,666 (95% UI: 10,796,198 - 29,302,986), with corresponding DALYs of 103,980 (95% UI: 38,249 - 249,703). Similarly, within female infertility, the highest cases and DALYs were also found in middle SDI regions, with figures of 39,038,802 (95% UI: 20,324,320 - 70,133,766) and 211,708 (95% UI: 75,088 - 517,044), respectively (**Supplementary Table S7**). Both reproductive-aged male and female infertility exhibited the highest prevalence rates and DALYs rates in high-middle SDI regions in 2021. Notably, between 1990 and 2021, male infertility had a rapid increase in both ASPR and ASDR in middle-low SDI regions, with EAPC of 1.00 (95% UI: 0.61 - 1.40) and 0.95 (95% UI: 0.57 - 1.33), respectively. In contrast, female infertility displayed a swift increase in ASPR and ASDR in high SDI regions, with EAPC values of 1.43 (95% UI: 1.28 - 1.58) and 1.41 (95% UI: 1.25 - 1.56), respectively. In fact, female infertility exhibited EAPC values > 0 across all five SDI regions analyzed. However, in low SDI regions, male infertility recorded a downward trend in both ASPR and ASDR from 1990 to 2021, with EAPC values of -0.17 (95% UI: -0.45 - 0.12) and -0.16 (95% UI: -0.44 - 0.12) (**Supplementary Table S7**). Among 21 regions, a negative correlation was found between disease burden and SDI when the SDI ranged from 0 to 0.4 and from 0.7 to 1, while greater volatility occurred in the 0.4 to 0.7 range. Eastern Europe and East Asia showed higher infertility burdens than expected, whereas Andean Latin America and Australasia exhibited lower-than-expected burdens (**Figures 4A, B**, **Supplementary Figures S4A, B**). Across 204 countries and regions, the overall burden of infertility for both males and females demonstrated a negative correlation, indicating that disease burden decreased as economic conditions improved. However, minimal fluctuations were observed within an SDI range of 0.5 to 0.75. In certain countries, such as Cameroon, the Central African Republic, Djibouti, Gabon, and the Russian Federation, the burden of infertility exceeded expectations; conversely, countries like Colombia, Australia, Burundi, and Malawi had infertility burdens that were lower than anticipated (**Figures 4C, D**, **Supplementary Figures S4C, D**).

### Temporal joinpoint analysis

**Figure 5:**
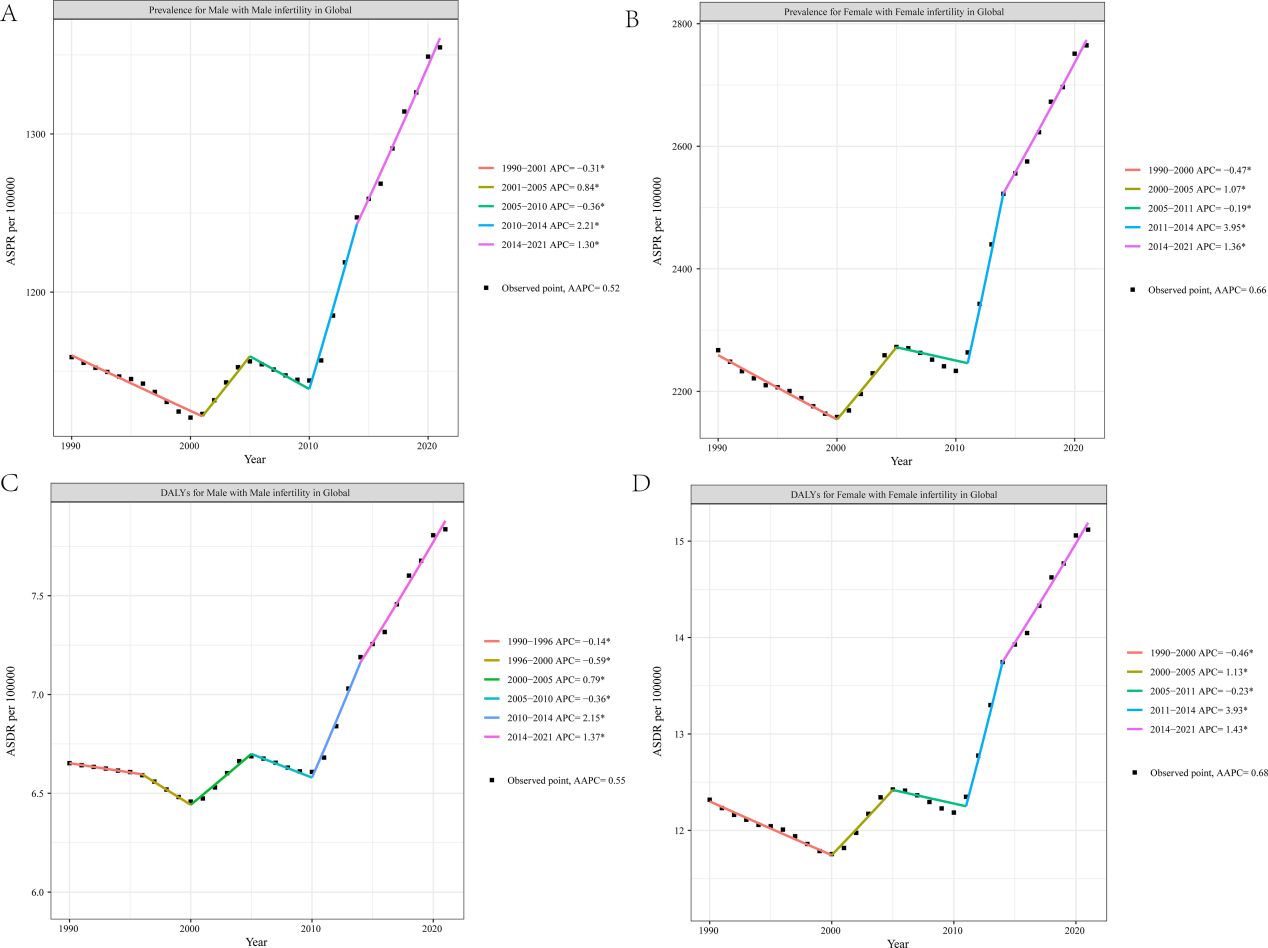
Joinpoint regression analysis of the male and female fertility burden trends, 1990–2021. Age-standardized prevalence rates for male infertility **(A)** and female infertility **(B)**; age-standardized disability-adjusted life-years rates for male infertility **(C)** and female infertility **(D)**. ASPR, age-standardized prevalence rate; ASDR, age-standardized disability-adjusted life-year rate.

Joinpoint regression analysis indicated that from 1990 to 2021, the ASPR for reproductive-aged male and female infertility exhibited an overall increasing trend, with an AAPC of 0.52% (95% CI: 0.43 - 0.60; P < 0.001) for males and 0.66% (95% CI: 0.57 to 0.76; P < 0.001) for females (**Figures 5A, B**, **Supplementary Table S8**). This suggested an annual increase in male infertility prevalence of 0.52% and 0.66% for females over the past 32 years. The Joinpoint regression model segmented the observation period for male infertility into five intervals: 1990-2001, 2001-2005, 2005-2010, 2010-2014, and 2014-2021. During the intervals of 1990–2001 and 2005-2010, the male infertility ASPR demonstrated a declining trend, with APC of -0.31 (95% CI: -0.36 to -0.25; P < 0.001) and -0.36 (95% CI: -0.61 to -0.11; P = 0.007), respectively. Notably, the most pronounced increase was observed in the 2010–2014 interval, with an APC of 2.21 (95% CI: 1.81 to 2.61; P < 0.001). The analysis for female infertility showed slightly different temporal intervals: 1990-2000, 2000-2005, 2005-2011, 2011-2014, and 2014-2021. However, the trend was consistent with male infertility. The changes in ASDR for both genders reflect the trends in prevalence rates, with both showing an overall increase from 1990 to 2021 (AAPC = 0.55; 95% CI: 0.45 to 0.64; P < 0.001) for males and (AAPC = 0.68; 95% CI: 0.58 to 0.78; P < 0.001) for females (**Figures 5C, D**, **Supplementary Table S8**).

Subsequently, a regional analysis was conducted. In Andean Latin America, both ASPR and ASDR began to rise starting in 1995, with the most rapid increases observed between 2019 and 2021: (AAPC = 12.66; 95% CI: 9.16 to 16.27; P < 0.001) for males and (AAPC = 52.89; 95% CI: 31.62 to 77.6; P < 0.001) for females. Conversely, Southern Sub-Saharan Africa experienced the fastest decline in ASPR and ASDR during the 2011–2015 period, with values of (AAPC = -10.89; 95% CI: -12.55 to -9.2; P < 0.001) and (AAPC = -12.98; 95% CI: -15.8 to -10.06; P < 0.001), respectively. It is noteworthy that, among males, Andean Latin America, Central Latin America, and Western Europe have shown an upward trend in both ASPR and ASDR since the 1990s, whereas East Asia and Oceania displayed a downward trend. In females, rising trends in ASPR and ASDR were observed in Andean Latin America, Central Europe, Central Latin America, and Eastern Europe since the 1990s, while East Asia exhibited a declining trend (**Supplementary Tables S9–S12**).

## Discussion

Infertility represents a significant burden for many families, impacting both individual and public health, and has emerged as a major public health challenge worldwide. This study was a comprehensive assessment of the burden of reproductive-aged infertility globally, as well as across 21 regions and 204 countries, utilizing the latest data from the GBD 2021. Previous research on the burden of infertility had not provided a thorough overview; thus, this study filled an important gap. Our research offered a detailed estimate of the prevalence and DALYs associated with infertility in both sexes of reproductive age. Furthermore, we employed the EAPC and Joinpoint regression model to analyze trends from 1990 to 2021. First, we found that from 1990 to 2021, the ASPR and ASDR for infertility among both sexes exhibited an overall upward trend. Second, except for Andean Latin America, Australasia, High-income Asia Pacific, and High-income North America, the prevalence of female infertility exceeded that of male infertility in both global and 17 additional regions, with a more pronounced growth trend for females compared to males. Third, the highest prevalence and DALYs for infertility among both sexes were observed in the 35–39 age group. Finally, the regions with high SDI recorded the lowest ASPR and ASDR, and a negative correlation was identified between infertility rates and SDI for regions with an SDI ranged from 0 to 0.4 and from 0.7 to 1; the burden of infertility across the 204 analyzed countries and regions demonstrated a negative correlation with SDI.

In 2021, the estimated global cases of infertility among both sexes were approximately 165,090,266. However, this result should be interpreted with caution due to significant uncertainty surrounding these estimates. In many low-economy countries, limitations in healthcare infrastructure may contribute to high rates of misdiagnosis and underdiagnosis, along with poorly developed disease reporting systems. In both developed and developing countries, only about half of individuals experiencing fertility issues pursued reproductive healthcare services (12). Many chose to refrain from seeking assistance because they hoped to conceive naturally or believed they did not have a fertility problem (9, 20). These factors may also contribute to the unreliability of current data. Our findings indicated that from 1990 to 2021, the ASPR and ASDR for infertility among women of reproductive age exhibited an upward trend globally, with a higher proportion of cases occurring among females. This observation aligned with previous studies, underscoring the necessity for heightened attention to the burden of infertility in women (10). Several factors may contribute to the higher prevalence of female infertility. The female reproductive system is inherently more complex, involving multiple organs such as the uterus, ovaries, and fallopian tubes, all of which play critical roles in fertility. In contrast, the male reproductive system is comparatively simpler, primarily comprising the testes, epididymis, and vas deferens. Consequently, women are more susceptible to infertility due to issues arising at various stages within their reproductive systems. Additionally, the quantity and quality of a woman’s oocytes decrease with age, particularly after the age of 35, leading to a sharp decline in fertility. While male sperm count and viability are also affected by age, the changes tend to occur at a more gradual pace. In addition to organic causes of infertility, factors such as stress, environmental pollution, and unhealthy dietary habits prevalent in modern life had adversely impacted fertility, further exacerbating infertility issues (21). High-income countries have shifted from traditional dietary practices to western dietary patterns, characterized by increased consumption of processed foods, high sugar intake, and the incorporation of trans fatty acids (22). However, such dietary regimens were often considered detrimental, being associated with numerous health conditions and weight gain (23). Both obesity and being overweight have been recognized as factors that impair fertility in both women and men (24). Animal studies have linked this western diet to lower progesterone levels, compromised endometrial function, and reduced semen concentration (25–28). A balanced diet rich in essential nutrients, vitamins, and minerals was vital for reproductive health in both men and women (29). Adopting a healthy lifestyle that including nutritious eating, regular exercise, and effective stress management can significantly improve fertility outcomes for individuals or couples trying to conceive (30). Furthermore, environmental factors and internal influences, such as pollution and endocrine disorders, can more significantly impact oocyte quality, thereby increasing the risk of infertility. Endocrine disorders can be a potential barrier to fertility, and detrimental lifestyle habits—such as prolonged late nights, irregular eating patterns, and lack of exercise—exert a more pronounced negative effect on women. Psychological stress can also disrupt the endocrine system, further complicating fertility issues.

Our research found that, from 1990 to 2021, among individuals aged 15 to 49 globally, the highest prevalence of infertility was observed in the 35–39 age group, while the lowest prevalence occurred in the 15–19 age group. Cross-sectional population surveys indicated that women who gave birth for the first time at age 35 or older had significantly higher age-adjusted rates of infertility compared to those who first gave birth before age 25 (31). In recent decades, many individuals have chosen to delay marriage and childbirth due to changing modern lifestyles. As age increases, women experienced a gradual decline in fertility, which may exacerbate infertility issues (32). The definition of infertility is closely related to the intention to conceive. In women over 40 years of age, the desire for pregnancy often declines, and regular use of contraception may obscure the diagnosis of infertility, making it challenging to assess their true reproductive status. This phenomenon may account for the observed decrease in infertility prevalence among the 40–49 age group.

Reproductive-aged male and female infertility was most prevalent in middle SDI regions, accounting for approximately one-third of the global total, and its prevalence rates were on the rise. Conversely, high SDI regions reported the lowest number of cases and ASPR for infertility. This trend may be attributed to the availability of advanced medical resources. However, it was important to note that these high-SDI areas exhibited the highest EAPC, indicating that they may face a significant burden of infertility in the future. This apparent discrepancy may be explained by a combination of socioeconomic and healthcare-related factors. In high SDI countries, delayed childbearing due to extended education and career development has become increasingly common, contributing to higher rates of age-related infertility in recent years (33, 34). Additionally, lifestyle risk factors such as obesity, sedentary behavior, smoking, and psychological stress—which are more prevalent in urban, high-income settings—may also play a role in the rising infertility rates. Moreover, high SDI regions typically have better access to healthcare and advanced diagnostic tools, leading to higher detection rates of infertility, particularly for subclinical or previously undiagnosed cases (12). Increased public awareness, reduced stigma, and a growing demand for fertility services may further encourage early medical consultations and contribute to the observed increase in reported cases. In low SDI regions, the EAPC was the lowest, primarily since countries in Sub-Saharan Africa often report EAPCs of < 0. One representative country, Malawi, has demonstrated a marked decline in both ASPR and ASDR for infertility from 1990 to 2021. This suggested that the burden of infertility may be lower in certain African countries, potentially due to ethnic and cultural differences (11). Further investigation was warranted to explore these underlying factors. Socio-economic status has been well established as a significant determinant of reproductive health (35). Research has demonstrated a negative correlation between education levels and infertility, which may help explain the lower prevalence of infertility in high SDI regions (36). Higher educational attainment was typically associated with healthier lifestyles and better access to medical care (37). Moreover, couples in high-income brackets posed the highest risk for infertility compared to lower-income groups. High-income couples tended to delay childbirth and may have previous histories of induced abortion, which could further contribute to their increased risk of infertility (38).

Joinpoint regression analysis revealed that the trends in ASPR for infertility among both males and females exhibited four distinct inflection points. Initially, from 1990 to 2000-2001, there was a decreasing trend in infertility rates, which may be attributed to the implementation of family planning policies in two of the world’s most populous countries. India introduced one of the first family planning initiatives in 1951, while China implemented its family planning policy in 1978. These policies led to widespread contraceptive use, resulting in a potential decline in global infertility ASPR and ASDR during this period. However, from 2000–2001 to 2021, both ASPR and ASDR exhibited an overall upward trend. The early 21st century was marked by significant economic development and a faster pace of life, which may have contributed to an increase in mental and physical health issues among younger individuals. Additionally, economic growth has been linked to exacerbated global environmental pollution, which can further heighten the burden of infertility. As family planning policies have relaxed and advancements in medical technology have progressed, previously hidden infertility populations may become more apparent. Since the outbreak of the COVID-19 pandemic in December 2019, it has had a profound impact on both physical and mental health worldwide, and has increasingly been recognized as a multisystem disease affecting various organs. Studies have shown that women infected with COVID-19 may experience significant alterations in sex hormone levels, which can manifest as menstrual irregularities or, in some cases, infertility due to ovarian dysfunction or failure resulting from suppressed ovarian activity (39–42). A longitudinal follow-up study found that male fertility may be temporarily reduced for up to 60 days following COVID-19 infection; however, this finding requires further validation (43). In addition, during the pandemic, psychological factors such as stress, anxiety, and emotional distress may have contributed to an increased risk of infertility in both men and women. Although COVID-19 has had a widespread impact on global health systems and reproductive services, current data from the GBD study showed a declining trend in the prevalence and DALYs of infertility between 2020 and 2021 (**Figures 5A–D**). This may be partly due to the temporary reduction in access to infertility diagnosis and treatment services during the pandemic, reporting delays, and limited direct epidemiological evidences. Therefore, in the post-COVID-19 period, healthcare professionals should enhance early screening and assessment of reproductive-aged couples, and promote health education and public awareness on reproductive health.

## Limitation

However, it is important to acknowledge the limitations of this study. First, the results are entirely dependent on the data quality of the GBD 2021, which primarily compiles information from national and regional reports and publications rather than directly from country-specific research. This reliance may lead to issues related to data completeness and quality, thereby affecting the accuracy of the conclusions. This concern is particularly relevant in low-income regions, where access to original data may be limited, and insufficient medical resources can increase the likelihood of misdiagnosis and underdiagnosis. Furthermore, the GBD 2021 does not quantify the extent to which these biases influence global estimates. Second, while GBD modeling techniques aim to standardize data, these inherent limitations can still impact the accuracy and comparability of the findings. Second, it should be noted that the sources of GBD data do not encompass all populations or regions; therefore, the findings only represent a general overview of specific areas. Third, GBD does not provide an analysis of risk factors related to infertility, limiting our ability to compare the magnitude of various infertility risk factors. More importantly, GBD2021 do not provide a burden of morbidity for infertility. Lastly, the study does not specify the causes of infertility.

## Conclusion

In summary, this research utilized GBD 2021 data to assess the burden of reproductive-aged infertility among both sexes globally, regionally, and nationally, analyzing trends from 1990 to 2021. The results indicate a significant increase in the burden of infertility among individuals aged 15–49 over the past 32 years, influenced by multiple factors, thereby representing a global public health challenge. Our findings provide essential evidence for evaluating epidemiological trends and formulating more effective national health policies, while also highlighting the critical need for continual improvements in infertility diagnosis, treatment, and management policies.

## Data Availability

The original contributions presented in the study are included in the article/**Supplementary Material**. Further inquiries can be directed to the corresponding authors.
