# Supplementary material for: Global, regional, and national burden and trends of reproductive-aged male and female infertility from 1990–2021
Source: Front Endocrinol (Lausanne). 2025 Sep 4;16:1506229. doi: 10.3389/fendo.2025.1506229 (PMC12443578; doi:10.3389/fendo.2025.1506229)
Supplement: Supplementary file 1 [file DataSheet1.docx]

**Table S1** Prevalence and DALYs for infertility in 21 regions in 2021

| **Location** | **Prevalence (95% UI)** | | **DALYs (95% UI)** | |
| --- | --- | --- | --- | --- |
|  | **Male** | **Female** | **Male** | **Female** |
| Andean Latin America | 232,367 (128,537 - 402,807) | 180,844 (18,126 - 574,607) | 1,308 (457 - 3,074) | 970 (83 - 3,433) |
| Australasia | 94,399 (53,292 - 163,037) | 23,946 (5,824 - 97,727) | 566 (208 -1,403) | 135 (22 - 593) |
| Caribbean | 320,709 (202,005 - 504,649) | 589,496 (342,122 - 997,994) | 1,865 (701 - 4,268) | 3,230 (1,194 - 8,225) |
| Central Asia | 482,054 (278,127 - 809,038) | 798,112 (353,395 - 1,591,761) | 2,798 (1,071 - 6,651) | 4,353 (1,393 - 10,832) |
| Central Europe | 751,761 (408,846 - 1,337,049) | 1,334,989 (617,168 - 2,521,006) | 4,209 (1,490 - 10,604) | 7,124 (2,268 - 18,324) |
| Central Latin America | 1,366,956 (804,646 - 2,251,065) | 2,270,205 (933,287 - 4,395,051) | 7,737 (2,830 - 17,728) | 12,076 (3,458 - 32,018) |
| Central Sub-Saharan Africa | 842,776 (472,132 - 1,354,804) | 1,705,616 (872,692 - 3,067,605) | 4,740 (1,668 - 11,334) | 9,141 (2,975 - 21,605) |
| East Asia | 12,167,667 (6,670,101 - 21,239,731) | 30,097,127 (14,985,652 - 53,555,149) | 65,671 (22,295 - 160,100) | 157,326 (51,989 - 406,568) |
| Eastern Europe | 2,041,839 (1,134,417 - 3,432,078) | 3,640,758 (1,828,022 - 6,714,993) | 11,889 (4,220 - 29,847) | 20,006 (6,812 - 52,654) |
| Eastern Sub-Saharan Africa | 1,481,393 (872,091 - 2,357,013) | 3,960,451 (2,299,441 - 6,526,195) | 8,420 (3,013 - 19,823) | 21,486 (7,752 - 50,131) |
| High-income Asia Pacific | 797,761 (429,920 - 1,382,800) | 555,824 (51,072 - 1,751,423) | 4,516 (1,588 - 11,264) | 2,963 (191 - 11,210) |
| High-income North America | 1,752,539 (954,878 - 2,959,535) | 1,478,922 (218,891 - 3,682,781) | 10,826 (3,847 - 26,993) | 8,507 (1,021 - 26,836) |
| North Africa and Middle East | 4,150,567 (2,458,605 - 6,611,301) | 6,357,159 (3,075,148 - 10,825,896) | 25,132 (9,234 - 57,501) | 35,930 (11,008 - 89,848) |
| Oceania | 50,725 (35,213 - 72,464) | 75,092 (42,007 - 115,416) | 292 (110 - 676) | 416 (144 - 1,001) |
| South Asia | 15,223,186 (8,656,105 - 25,000,575) | 35,555,258 (19,863,521 - 60,335,282) | 90,312 (32,227 - 209,136) | 199,476 (71,920 - 470,290) |
| Southeast Asia | 5,370,095 (3,092,324 - 8,839,732) | 11,004,744 (5,574,141 - 19,376,109) | 31,369 (11,255 - 73,878) | 60,681 (21,015 - 150,690) |
| Southern Latin America | 361,275 (206,015 - 615,642) | 387,109 (94,046 - 864,584) | 2,142 (776 - 5,318) | 2,160 (370 - 6,141) |
| Southern Sub-Saharan Africa | 320,676 (168,458 - 598,876) | 782,508 (260,859 - 1,743,635) | 1,819 (619 - 4,609) | 4,197 (1,096 - 11,247) |
| Tropical Latin America | 1,384,604 (780,681 - 2,299,828) | 2,215,723 (943,705 - 4,309,763) | 8,117 (2,920 - 19,585) | 12,166 (3,807 - 32,977) |
| Western Europe | 1,933,592 (1,070,793 - 3,257,464) | 2,492,439 (748,783 - 5,485,462) | 11,857 (4,236 - 29,090) | 14,102 (3,264 - 39,770) |
| Western Sub-Saharan Africa | 3,873,874 (2,374,489 - 5,892,117) | 4,583,137 (2,061,353 - 8,923,482) | 22,028 (8,143 - 51,657) | 24,687 (7,276 - 61,455) |

DALYs, disability-adjusted life-years; UI, uncertainty interval.

**Table S2** ASPR and ASDR for infertility in 21 regions in 2021

| **Location** | **ASPR/100,000 (95% UI)** | | **ASDR/100,000 (95% UI)** | |
| --- | --- | --- | --- | --- |
|  | **Male** | **Female** | **Male** | **Female** |
| Andean Latin America | 655.75 (363.35 -1,142.51) | 504.63 (50.37 - 1,593.83) | 3.69 (1.29 - 8.74) | 2.71 (0.23 - 9.58) |
| Australasia | 622.98 (350.36 -1,073.96) | 152.74 (37.22 - 604.18) | 3.76 (1.37 - 9.32) | 0.87 (0.14 - 3.67) |
| Caribbean | 1,331.15 (838.46 - 2,090.09) | 2,406.71 (1,390.62 - 4,061.59) | 7.74 (2.89 - 17.77) | 13.19 (4.88 - 33.64) |
| Central Asia | 933.06 (541.89 - 1,535.07) | 1,540.98 (693.02 - 3,004.33) | 5.44 (2.08 - 12.78) | 8.44 (2.67 - 20.74) |
| Central Europe | 1,371.77 (748.4 - 2,424.41) | 2,528.33 (1,205.28 - 4,746.56) | 7.77 (2.74 - 19.83) | 13.6 (4.29 - 34.98) |
| Central Latin America | 1,049.57 (618.04 - 1,736.39) | 1,637.03 (676.33 - 3,179.79) | 5.93 (2.18 - 13.66) | 8.71 (2.5 - 23.2) |
| Central Sub-Saharan Africa | 1,411.72 (789.07 - 2,271.46) | 2,865.53 (1,445.2 - 5,372.44) | 7.86 (2.75 - 19.15) | 15.23 (4.7 - 35.74) |
| East Asia | 1,575.7 (880.94 - 2,675.46) | 4,102.68 (2,124.47 - 7,170.94) | 8.57 (2.94 - 20.87) | 21.55 (7.4 - 54.1) |
| Eastern Europe | 2,058.13 (1,120.2 - 3,444.23) | 3,604.24 (1,850.66 - 6,527.52) | 12.2 (4.27 - 30.22) | 20.11 (7.05 - 51.09) |
| Eastern Sub-Saharan Africa | 777.87 (459.72 - 1,236.42) | 1,966.79 (1,124.46 - 3,295.26) | 4.37 (1.59 - 10.17) | 10.57 (3.79 - 25.04) |
| High-income Asia Pacific | 898.57 (491.48 - 1,481.48) | 605.07 (61.02 - 1,892.11) | 5.17 (1.8 - 12.84) | 3.24 (0.24 - 12.55) |
| High-income North America | 1,010.31 (546.66 - 1,701.88) | 848.73 (128 - 2,124.44) | 6.26 (2.19 - 15.78) | 4.9 (0.58 - 15.3) |
| North Africa and Middle East | 1,154.32 (681.51 - 1,863.21) | 1,928.83 (931.82 - 3,266.23) | 7.02 (2.56 - 16.24) | 10.93 (3.36 - 27.16) |
| Oceania | 712.44 (498.04 - 1,011.41) | 1,068.87 (593.82 - 1,647.74) | 4.08 (1.55 - 9.43) | 5.9 (2.05 - 14.25) |
| South Asia | 1,464.33 (835.34 - 2,404.5) | 3,523.38 (1,963.94 - 5,994.84) | 8.67 (3.11 - 20.15) | 19.74 (7.09 - 46.79) |
| Southeast Asia | 1,399.74 (805.71 - 2,309.49) | 2,919.2 (1,477.11 - 5,150.66) | 8.19 (2.93 - 19.24) | 16.11 (5.57 - 40.33) |
| Southern Latin America | 1,014.36 (577.04 - 1,717.53) | 1,060.26 (260.27 - 2,339.84) | 6.02 (2.19 - 14.98) | 5.93 (1.01 - 17.1) |
| Southern Sub-Saharan Africa | 718.21 (382.24 - 1,327.36) | 1,715.36 (578.85 - 3,827.21) | 4.08 (1.39 - 10.21) | 9.21 (2.42 - 24.57) |
| Tropical Latin America | 1,110.32 (629.61 - 1,860.19) | 1,697 (729.23 - 3,288.91) | 6.53 (2.37 - 15.72) | 9.35 (3 - 25.33) |
| Western Europe | 972.43 (540.33 - 1,653.86) | 1,224.48 (367.65 - 2,644.73) | 6.03 (2.13 - 14.95) | 6.99 (1.63 - 19.39) |
| Western Sub-Saharan Africa | 1,918.52 (1,198.23 - 2,948.44) | 2,057.2 (894.15 - 4,060.81) | 10.78 (3.94 - 25.15) | 10.97 (3.17 - 27.28) |

ASPR, age-standardized prevalence rate; ASDR, age-standardized disability-adjusted life-years rate; UI, uncertainty interval

**Table S3** EAPC of ASPR and ASDR for infertility in 21 regions from 1990 to 2021

| **Location** | **EAPC of ASIR (95% CI)** | | **EAPC of ASDR (95% CI)** | |
| --- | --- | --- | --- | --- |
|  | **Male** | **Female** | **Male** | **Female** |
| Andean Latin America | 2.14 (1.77 - 2.51) | 8.22 (6.7 - 9.76) | 2.06 (1.72 - 2.4) | 8.11 (6.62 - 9.62) |
| Australasia | 0.25 (0.21 - 0.29) | 0.86 (0.71 - 1.01) | 0.26 (0.22 - 0.3) | 0.83 (0.69 - 0.97) |
| Caribbean | -0.09 (-0.2 - 0.01) | -0.11 (-0.28 - 0.06) | -0.12 (- 0.21 - -0.03) | -0.13 (-0.28 - 0.03) |
| Central Asia | 0.38 (0.24 - 0.53) | 0.89 (0.63 - 1.14) | 0.34 (0.21 - 0.48) | 0.83 (0.59 - 1.07) |
| Central Europe | 0.62 (0.51 - 0.73) | 0.88 (0.74 - 1.02) | 0.58 (0.48 - 0.68) | 0.84 (0.71 - 0.98) |
| Central Latin America | 0.51 (0.27 - 0.75) | 1.19 (0.8 - 1.58) | 0.49 (0.26 - 0.72) | 1.17 (0.79 - 1.55) |
| Central Sub-Saharan Africa | -0.38 (-0.79 - 0.04) | -0.17 (-0.78 - 0.45) | -0.35 (-0.75 - 0.05) | -0.13 (-0.73 - 0.47) |
| East Asia | 0 (-0.05 - 0.06) | 0.01 (-0.04 - 0.05) | 0.02 (-0.04 - 0.09) | 0.02 (-0.03 - 0.07) |
| Eastern Europe | 0.26 (0.15 - 0.36) | 0.6 (0.48 - 0.73) | 0.26 (0.16 - 0.37) | 0.58 (0.45 - 0.7) |
| Eastern Sub-Saharan Africa | -1.19 (-1.41 - -0.96) | -1.25 (-1.52 - -0.98) | -1.17 (-1.39 - -0.95) | -1.23 (-1.49 - -0.97) |
| High-income Asia Pacific | -0.2 (-0.29 - -0.11) | -0.39 (-0.65 - -0.13) | -0.19 (-0.27 - -0.11) | -0.4 (-0.65 - -0.15) |
| High-income North America | 0.83 (0.19 - 1.47) | 3.01 (1.61 - 4.43) | 0.76 (0.12 - 1.41) | 2.91 (1.51 - 4.33) |
| North Africa and Middle East | 0.8 (0.68 - 0.93) | 1.19 (0.98 - 1.39) | 0.73 (0.6 - 0.85) | 1.09 (0.88 - 1.3) |
| Oceania | -0.96 (-1.17 - -0.75) | -1.58 (-1.85 - -1.3) | -0.94 (-1.13 - -0.74) | -1.53 (-1.8 - -1.26) |
| South Asia | 1.52 (0.96 - 2.09) | 1.95 (1.2 - 2.71) | 1.43 (0.89 - 1.96) | 1.85 (1.13 - 2.57) |
| Southeast Asia | 1.59 (1.3 - 1.88) | 1.68 (1.34 - 2.02) | 1.55 (1.28 - 1.82) | 1.66 (1.33 - 1.98) |
| Southern Latin America | -0.05 (-0.12 - 0.03) | -0.25 (-0.33 - -0.16) | -0.04 (-0.1 - 0.03) | -0.26 (-0.34 - -0.18) |
| Southern Sub-Saharan Africa | -0.68 (-1.22 - -0.13) | -0.75 (-1.49 - 0) | -0.72 (-1.26 - -0.18) | -0.8 (-1.54 - -0.06) |
| Tropical Latin America | 1.83 (1.41 - 2.26) | 1.71 (1.13 - 2.29) | 1.76 (1.34 - 2.18) | 1.62 (1.05 - 2.21) |
| Western Europe | 0.95 (0.75 - 1.14) | 1.48 (1.2 - 1.76) | 0.93 (0.74 - 1.12) | 1.44 (1.17 - 1.72) |
| Western Sub-Saharan Africa | -0.62 (-0.88 - -0.36) | -0.32 (-0.6 - -0.04) | -0.6 (-0.87 - -0.34) | -0.3 (-0.57 - -0.03) |

ASPR, age-standardized prevalence rate; ASDR, age-standardized disability-adjusted life-years rate; CI, confidence interval; EAPC estimated annual percentage change

**Table S4** Prevalence and DALYs for infertility in 204 countries in 2021

| **Location** | **Prevalence (95% UI)** | | **DALYs (95% UI)** | |
| --- | --- | --- | --- | --- |
|  | **Male** | **Female** | **Male** | **Female** |
| Afghanistan | 82555 (60010 - 109613) | 103774 (65690 - 142851) | 475 (191 - 1045) | 567 (205 - 1284) |
| Albania | 9223 (6726 - 12695) | 12163 (8866 - 15886) | 55 (21 - 121) | 68 (26 - 139) |
| Algeria | 241720 (135725 - 390349) | 411463 (180229 - 736498) | 1445 (510 - 3349) | 2282 (642 - 5900) |
| American Samoa | 252 (142 - 400) | 450 (190 - 851) | 1 (1 - 3) | 2 (1 - 6) |
| Andorra | 374 (201 - 611) | 495 (139 - 1018) | 2 (1 - 6) | 3 (1 - 8) |
| Angola | 227759 (126022 - 367404) | 533943 (287662 - 941956) | 1282 (459 - 3063) | 2854 (982 - 6963) |
| Antigua and Barbuda | 711 (405 - 1152) | 1447 (740 - 2618) | 4 (1 - 10) | 8 (3 - 21) |
| Argentina | 244880 (139991 - 414873) | 261639 (63094 - 579074) | 1450 (527 - 3571) | 1465 (248 - 4055) |
| Armenia | 6683 (4701 - 9212) | 7690 (4384 - 11654) | 38 (15 - 83) | 41 (14 - 95) |
| Australia | 71316 (40257 - 123061) | 16214 (4511 - 69965) | 429 (158 - 1049) | 93 (16 - 412) |
| Austria | 71318 (39049 - 118115) | 126855 (56158 - 232867) | 425 (151 - 1056) | 697 (218 - 1691) |
| Azerbaijan | 77867 (42953 - 131396) | 135501 (56369 - 276168) | 458 (165 - 1131) | 747 (235 - 1999) |
| Bahamas | 3086 (1761 - 5125) | 6013 (3031 - 11082) | 18 (6 - 44) | 33 (11 - 84) |
| Bahrain | 15149 (8766 - 24343) | 12863 (5953 - 22979) | 91 (32 - 211) | 72 (21 - 183) |
| Bangladesh | 640268 (476969 - 849628) | 1506087 (1190413 - 1810954) | 3835 (1538 - 8178) | 8613 (3349 - 17060) |
| Barbados | 2060 (1172 - 3366) | 4146 (2113 - 7506) | 12 (4 - 30) | 23 (8 - 59) |
| Belarus | 64003 (35736 - 111882) | 115515 (50429 - 227190) | 375 (134 - 968) | 634 (197 - 1667) |
| Belgium | 76409 (42082 - 124951) | 135219 (58389 - 247336) | 460 (166 - 1139) | 750 (218 - 1844) |
| Belize | 2159 (1204 - 3578) | 3321 (1311 - 6349) | 13 (5 - 31) | 19 (5 - 47) |
| Benin | 62215 (46183 - 82438) | 43774 (17721 - 70639) | 351 (141 - 760) | 236 (72 - 617) |
| Bermuda | 408 (229 - 682) | 824 (404 - 1502) | 2 (1 - 6) | 5 (1 - 12) |
| Bhutan | 3411 (1975 - 5572) | 6049 (2539 - 11297) | 20 (7 - 49) | 34 (10 - 83) |
| Bolivia (Plurinational State of) | 39123 (21842 - 65206) | 24344 (2796 - 79116) | 221 (78 - 516) | 132 (10 - 510) |
| Bosnia and Herzegovina | 15762 (8386 - 28127) | 28239 (11725 - 59026) | 88 (31 - 218) | 151 (43 - 389) |
| Botswana | 11385 (5882 - 21210) | 32629 (12794 - 67450) | 62 (21 - 154) | 170 (47 - 457) |
| Brazil | 1335417 (751579 - 2223673) | 2125447 (887052 - 4170154) | 7834 (2808 - 18813) | 11672 (3616 - 31644) |
| Brunei Darussalam | 2103 (1153 - 3659) | 952 (109 - 3398) | 12 (4 - 30) | 5 (0 - 22) |
| Bulgaria | 32135 (16713 - 58437) | 56192 (22571 - 120187) | 178 (61 - 448) | 297 (84 - 747) |
| Burkina Faso | 146018 (86240 - 236973) | 151357 (46038 - 322972) | 824 (296 - 1929) | 813 (173 - 2240) |
| Burundi | 17048 (12392 - 22593) | 20729 (11395 - 31123) | 96 (38 - 213) | 114 (39 - 279) |
| Cabo Verde | 7445 (4310 - 12108) | 8535 (3439 - 16585) | 42 (15 - 98) | 45 (13 - 108) |
| Cambodia | 89476 (49834 - 156018) | 220334 (102200 - 415498) | 498 (173 - 1207) | 1172 (367 - 2926) |
| Cameroon | 476601 (273224 - 762810) | 549482 (278289 - 975463) | 2789 (1020 - 6580) | 3034 (983 - 8142) |
| Canada | 110319 (59785 - 183430) | 81972 (8231 - 216141) | 691 (242 - 1692) | 482 (38 - 1589) |
| Central African Republic | 66253 (38820 - 100975) | 148169 (90817 - 237833) | 382 (142 - 909) | 817 (303 - 1882) |
| Chad | 129917 (74587 - 210510) | 143985 (52871 - 287190) | 711 (251 - 1619) | 756 (191 - 1785) |
| Chile | 99916 (55869 - 170472) | 106501 (24098 - 238349) | 593 (218 - 1474) | 590 (102 - 1740) |
| China | 11845804 (6488726 - 20756171) | 29317000 (14569167 - 52098692) | 63931 (21752 - 155614) | 153252 (50580 - 396547) |
| Colombia | 107619 (80964 - 144248) | 58268 (13249 - 111088) | 668 (269 - 1401) | 345 (62 - 920) |
| Comoros | 6765 (3756 - 11000) | 17948 (10106 - 28707) | 41 (14 - 96) | 102 (36 - 242) |
| Congo | 37161 (20611 - 62133) | 87057 (41048 - 161490) | 203 (72 - 484) | 453 (135 - 1181) |
| Cook Islands | 76 (44 - 121) | 178 (79 - 327) | 0 (0 - 1) | 1 (0 - 3) |
| Costa Rica | 26724 (14978 - 45397) | 49838 (20850 - 100255) | 153 (56 - 367) | 269 (74 - 740) |
| Coted'Ivoire | 360979 (215464 - 572863) | 405916 (192551 - 762142) | 2009 (752 - 4673) | 2148 (646 - 5223) |
| Croatia | 20164 (10641 - 36518) | 35645 (14306 - 77357) | 113 (39 - 278) | 190 (54 - 471) |
| Cuba | 77789 (44843 - 130879) | 144318 (74438 - 263135) | 456 (167 - 1082) | 792 (265 - 2049) |
| Cyprus | 6600 (3461 - 11333) | 9763 (2994 - 20762) | 41 (13 - 99) | 55 (13 - 159) |
| Czechia | 66984 (35828 - 117081) | 128037 (58522 - 245085) | 369 (129 - 926) | 677 (210 - 1769) |
| Democratic People's Republic of Korea | 178265 (97829 - 315800) | 410661 (201844 - 776717) | 964 (325 - 2334) | 2149 (676 - 5064) |
| Democratic Republic of the Congo | 479436 (267987 - 769452) | 862739 (409645 - 1644018) | 2691 (917 - 6442) | 4624 (1375 - 11278) |
| Denmark | 11994 (6025 - 21259) | 8206 (718 - 22725) | 79 (27 - 196) | 49 (4 - 161) |
| Djibouti | 13338 (7675 - 21473) | 36059 (20121 - 58772) | 75 (27 - 176) | 193 (68 - 471) |
| Dominica | 528 (302 - 876) | 929 (474 - 1730) | 3 (1 - 7) | 5 (2 - 13) |
| Dominican Republic | 77529 (44175 - 132196) | 139681 (63324 - 270113) | 450 (161 - 1095) | 763 (242 - 2010) |
| Ecuador | 59499 (32938 - 99141) | 34628 (4370 - 118287) | 339 (119 - 818) | 187 (15 - 732) |
| Egypt | 463785 (260526 - 751492) | 989412 (442270 - 1672172) | 2864 (990 - 6650) | 5698 (1652 - 13987) |
| El Salvador | 17663 (9688 - 30302) | 26372 (4332 - 64834) | 102 (36 - 245) | 143 (19 - 427) |
| Equatorial Guinea | 14032 (7925 - 22764) | 26891 (14679 - 47461) | 80 (28 - 190) | 143 (48 - 344) |
| Eritrea | 53372 (30039 - 87700) | 142798 (79663 - 237797) | 308 (109 - 729) | 783 (276 - 1807) |
| Estonia | 8784 (4880 - 15497) | 15140 (6954 - 28747) | 51 (19 - 130) | 83 (28 - 216) |
| Eswatini | 3358 (1766 - 5957) | 8002 (2446 - 17221) | 19 (6 - 47) | 44 (10 - 126) |
| Ethiopia | 409621 (229972 - 665447) | 992154 (476708 - 1804689) | 2347 (825 - 5537) | 5419 (1812 - 12945) |
| Fiji | 7359 (4276 - 11587) | 15293 (8060 - 26195) | 43 (15 - 103) | 85 (28 - 205) |
| Finland | 13778 (7204 - 23567) | 21050 (5178 - 45662) | 85 (27 - 203) | 120 (22 - 348) |
| France | 335291 (184957 - 570129) | 625853 (259355 - 1114550) | 2130 (722 - 4990) | 3662 (1064 - 9306) |
| Gabon | 18135 (10518 - 28533) | 46817 (26562 - 77282) | 103 (37 - 244) | 251 (85 - 620) |
| Gambia | 23548 (13978 - 37413) | 28494 (11775 - 55242) | 134 (48 - 306) | 152 (45 - 379) |
| Georgia | 17589 (9869 - 31477) | 26627 (9016 - 57764) | 101 (37 - 244) | 144 (37 - 372) |
| Germany | 214861 (113143 - 378600) | 220847 (38085 - 558906) | 1331 (445 - 3303) | 1244 (166 - 4044) |
| Ghana | 365821 (213465 - 601594) | 377924 (137289 - 822057) | 2056 (716 - 4728) | 1996 (473 - 5006) |
| Greece | 38226 (19901 - 64124) | 51813 (11610 - 113114) | 233 (79 - 576) | 288 (52 - 799) |
| Greenland | 188 (100 - 313) | 132 (13 - 356) | 1 (0 - 3) | 1 (0 - 3) |
| Grenada | 845 (473 - 1399) | 1439 (743 - 2680) | 5 (2 - 12) | 8 (3 - 20) |
| Guam | 827 (476 - 1331) | 1400 (539 - 2632) | 5 (2 - 11) | 8 (2 - 19) |
| Guatemala | 72757 (39985 - 121592) | 133608 (51054 - 283637) | 404 (143 - 928) | 706 (193 - 1920) |
| Guinea | 107894 (80108 - 142051) | 136365 (87441 - 189499) | 607 (240 - 1341) | 732 (265 - 1673) |
| Guinea-Bissau | 20131 (11833 - 31704) | 21954 (8174 - 42574) | 113 (42 - 261) | 118 (31 - 288) |
| Guyana | 7795 (4423 - 12410) | 15888 (8842 - 27705) | 46 (16 - 106) | 87 (30 - 222) |
| Haiti | 67852 (49964 - 88138) | 113714 (84161 - 142353) | 387 (152 - 829) | 619 (239 - 1340) |
| Honduras | 47706 (26010 - 82791) | 95108 (38407 - 188330) | 268 (93 - 634) | 505 (143 - 1393) |
| Hungary | 44970 (23697 - 80372) | 80012 (33659 - 166650) | 251 (88 - 621) | 426 (125 - 1080) |
| Iceland | 1503 (796 - 2533) | 1956 (557 - 4219) | 9 (3 - 23) | 11 (3 - 31) |
| India | 12352775 (7079212 - 20281847) | 29075289 (16070794 - 49483699) | 72582 (26125 - 167575) | 161474 (57797 - 392596) |
| Indonesia | 3096051 (1794084 - 5069623) | 6251542 (3293414 - 11133561) | 17850 (6496 - 43630) | 34094 (11773 - 82100) |
| Iran (Islamic Republic of) | 629495 (349767 - 1059011) | 815742 (367319 - 1472829) | 4098 (1426 - 9407) | 4978 (1502 - 14117) |
| Iraq | 255679 (143726 - 421404) | 385454 (183787 - 661420) | 1543 (540 - 3496) | 2166 (681 - 5349) |
| Ireland | 19521 (10196 - 33175) | 30327 (9074 - 64436) | 119 (41 - 282) | 169 (38 - 470) |
| Israel | 55542 (29795 - 93816) | 39816 (9916 - 90331) | 340 (116 - 847) | 223 (42 - 653) |
| Italy | 336010 (183301 - 573362) | 281020 (34316 - 762230) | 2008 (721 - 4873) | 1545 (168 - 5227) |
| Jamaica | 27346 (15710 - 44148) | 54434 (29719 - 95285) | 161 (58 - 372) | 301 (101 - 748) |
| Japan | 580359 (314229 - 992733) | 424422 (37954 - 1273566) | 3279 (1146 - 8086) | 2258 (142 - 8592) |
| Jordan | 71049 (51651 - 97108) | 99625 (74090 - 127976) | 434 (172 - 947) | 558 (214 - 1229) |
| Kazakhstan | 99020 (54670 - 173589) | 175036 (68958 - 370779) | 573 (211 - 1372) | 955 (268 - 2394) |
| Kenya | 231805 (131188 - 392200) | 617522 (314620 - 1122348) | 1295 (449 - 3187) | 3309 (1112 - 8149) |
| Kiribati | 637 (362 - 1037) | 1388 (632 - 2564) | 4 (1 - 9) | 8 (2 - 19) |
| Kuwait | 39121 (22284 - 64162) | 62231 (27749 - 113601) | 231 (85 - 543) | 342 (97 - 861) |
| Kyrgyzstan | 44537 (24858 - 76492) | 85510 (40458 - 163876) | 255 (94 - 601) | 462 (152 - 1133) |
| Lao People's Democratic Republic | 36276 (20152 - 62014) | 78106 (34643 - 147647) | 210 (73 - 508) | 428 (124 - 1100) |
| Latvia | 11991 (6569 - 21227) | 21562 (9796 - 41262) | 69 (24 - 175) | 117 (37 - 301) |
| Lebanon | 44170 (25517 - 72503) | 86054 (42323 - 153793) | 260 (92 - 608) | 468 (144 - 1127) |
| Lesotho | 10000 (5527 - 16731) | 28597 (13669 - 52989) | 57 (20 - 142) | 154 (50 - 404) |
| Liberia | 75567 (44328 - 118651) | 87235 (40516 - 163086) | 417 (150 - 979) | 456 (132 - 1138) |
| Libya | 45094 (25379 - 72492) | 69077 (30788 - 125656) | 274 (97 - 630) | 389 (109 - 1006) |
| Lithuania | 17650 (9684 - 31176) | 32232 (15148 - 62109) | 101 (35 - 247) | 174 (56 - 455) |
| Luxembourg | 2935 (1574 - 5064) | 3592 (905 - 8029) | 18 (6 - 46) | 20 (4 - 57) |
| Madagascar | 136438 (76999 - 217070) | 355631 (185469 - 597454) | 808 (283 - 1950) | 2003 (688 - 4801) |
| Malawi | 26259 (18466 - 35965) | 41567 (25927 - 57043) | 144 (56 - 311) | 224 (82 - 524) |
| Malaysia | 164290 (90291 - 286851) | 407011 (162381 - 812003) | 917 (314 - 2276) | 2151 (600 - 5596) |
| Maldives | 8044 (6116 - 10486) | 9189 (7308 - 11222) | 49 (19 - 103) | 52 (21 - 107) |
| Mali | 141754 (105273 - 187908) | 109179 (60461 - 158688) | 820 (320 - 1762) | 613 (203 - 1366) |
| Malta | 1852 (969 - 3115) | 2516 (762 - 5322) | 11 (4 - 27) | 14 (3 - 38) |
| Marshall Islands | 342 (198 - 556) | 634 (294 - 1154) | 2 (1 - 5) | 3 (1 - 9) |
| Mauritania | 49696 (29190 - 80746) | 58064 (26277 - 107048) | 290 (104 - 697) | 320 (95 - 894) |
| Mauritius | 5735 (3156 - 9638) | 12586 (4867 - 24531) | 33 (12 - 79) | 68 (19 - 172) |
| Mexico | 925251 (526665 - 1564207) | 1614547 (684946 - 3192541) | 5182 (1861 - 12018) | 8537 (2505 - 22628) |
| Micronesia (Federated States of) | 572 (332 - 920) | 1040 (480 - 1889) | 3 (1 - 8) | 6 (2 - 14) |
| Monaco | 116 (63 - 198) | 172 (50 - 351) | 1 (0 - 2) | 1 (0 - 3) |
| Mongolia | 18533 (10420 - 32374) | 31156 (12719 - 62694) | 106 (39 - 254) | 169 (50 - 444) |
| Montenegro | 3105 (1654 - 5465) | 5381 (2228 - 11408) | 17 (6 - 43) | 29 (8 - 75) |
| Morocco | 261111 (146773 - 422280) | 430242 (216983 - 739243) | 1603 (561 - 3828) | 2461 (749 - 6122) |
| Mozambique | 180570 (102126 - 295557) | 574163 (323719 - 930604) | 1029 (368 - 2475) | 3094 (1102 - 7419) |
| Myanmar | 158643 (118583 - 208422) | 296755 (201457 - 389740) | 941 (369 - 1959) | 1650 (620 - 3742) |
| Namibia | 7575 (4117 - 13324) | 19808 (6571 - 42758) | 43 (15 - 107) | 107 (27 - 290) |
| Nauru | 59 (33 - 96) | 119 (54 - 220) | 0 (0 - 1) | 1 (0 - 2) |
| Nepal | 73946 (53891 - 98753) | 150430 (109902 - 194827) | 460 (182 - 968) | 891 (344 - 1880) |
| Netherlands | 62800 (33223 - 105631) | 88185 (25914 - 189032) | 389 (136 - 954) | 499 (121 - 1365) |
| New Zealand | 23083 (12824 - 39575) | 7732 (1326 - 29990) | 137 (49 - 336) | 43 (5 - 180) |
| Nicaragua | 23350 (12756 - 39783) | 32873 (8145 - 74925) | 135 (48 - 308) | 178 (33 - 530) |
| Niger | 191421 (110870 - 302638) | 231243 (98432 - 427363) | 1099 (387 - 2500) | 1247 (349 - 2931) |
| Nigeria | 1423329 (813990 - 2299821) | 1892833 (719710 - 3940193) | 8103 (2822 - 19538) | 10217 (2610 - 26220) |
| Niue | 8 (4 - 13) | 16 (7 - 30) | 0 (0 - 0) | 0 (0 - 0) |
| North Macedonia | 12347 (6579 - 21911) | 21438 (8983 - 45206) | 69 (24 - 169) | 114 (34 - 291) |
| Northern Mariana Islands | 272 (152 - 433) | 433 (192 - 835) | 2 (1 - 4) | 2 (1 - 6) |
| Norway | 35280 (19315 - 59320) | 46117 (14503 - 93940) | 216 (78 - 528) | 261 (63 - 700) |
| Oman | 49144 (28475 - 80117) | 43442 (19846 - 76751) | 292 (106 - 685) | 240 (69 - 588) |
| Pakistan | 2152785 (718884 - 4603396) | 4817403 (1352930 - 10740041) | 13416 (3329 - 38643) | 28463 (5747 - 79955) |
| Palau | 117 (65 - 188) | 161 (66 - 305) | 1 (0 - 2) | 1 (0 - 2) |
| Palestine | 29382 (16678 - 48394) | 48573 (23815 - 84700) | 179 (63 - 421) | 274 (80 - 642) |
| Panama | 25289 (14097 - 43403) | 45747 (19728 - 90415) | 141 (51 - 325) | 242 (73 - 633) |
| Papua New Guinea | 31126 (22472 - 42814) | 37489 (20998 - 55295) | 178 (68 - 387) | 208 (67 - 519) |
| Paraguay | 49188 (27658 - 79438) | 90276 (48834 - 157990) | 283 (103 - 664) | 495 (169 - 1243) |
| Peru | 133745 (72437 - 238820) | 121873 (10651 - 372425) | 749 (254 - 1811) | 651 (48 - 2457) |
| Philippines | 1214217 (494659 - 2295376) | 2499071 (851174 - 4963466) | 7378 (2204 - 20024) | 14334 (3812 - 35154) |
| Poland | 365295 (205782 - 629519) | 657230 (324241 - 1192629) | 2054 (739 - 5290) | 3522 (1145 - 8861) |
| Portugal | 36656 (19446 - 60639) | 55230 (15934 - 118061) | 224 (76 - 548) | 309 (73 - 821) |
| Puerto Rico | 21371 (12086 - 34576) | 42735 (21025 - 78105) | 125 (46 - 303) | 235 (79 - 603) |
| Qatar | 42210 (23899 - 69306) | 24658 (10605 - 45057) | 251 (91 - 591) | 136 (38 - 333) |
| Republic of Korea | 189238 (100372 - 329317) | 112151 (10787 - 388446) | 1079 (371 - 2674) | 602 (41 - 2520) |
| Republic of Moldova | 30679 (16924 - 53007) | 52438 (24238 - 98104) | 180 (65 - 453) | 290 (95 - 741) |
| Romania | 88777 (46659 - 159439) | 150446 (61535 - 319646) | 495 (174 - 1232) | 799 (227 - 2108) |
| Russian Federation | 1495203 (820471 - 2534415) | 2715415 (1370335 - 5019099) | 8643 (3024 - 21559) | 14835 (5113 - 38356) |
| Rwanda | 52212 (29380 - 88025) | 46497 (11842 - 108671) | 301 (103 - 744) | 253 (48 - 703) |
| Saint Kitts and Nevis | 498 (278 - 838) | 945 (482 - 1695) | 3 (1 - 7) | 5 (2 - 13) |
| Saint Lucia | 1468 (828 - 2421) | 2726 (1358 - 5025) | 9 (3 - 21) | 15 (5 - 38) |
| Saint Vincent and the Grenadines | 879 (496 - 1440) | 1608 (800 - 2958) | 5 (2 - 12) | 9 (3 - 23) |
| Samoa | 1021 (591 - 1670) | 1945 (846 - 3639) | 6 (2 - 14) | 11 (3 - 26) |
| San Marino | 109 (58 - 183) | 166 (48 - 355) | 1 (0 - 2) | 1 (0 - 3) |
| Sao Tome and Principe | 1381 (796 - 2217) | 1155 (275 - 2799) | 8 (3 - 18) | 6 (1 - 17) |
| Saudi Arabia | 366476 (209758 - 594948) | 432939 (194918 - 767797) | 2173 (769 - 5053) | 2393 (684 - 5944) |
| Senegal | 120189 (91989 - 156117) | 117792 (73765 - 167570) | 703 (278 - 1534) | 644 (234 - 1431) |
| Serbia | 46846 (24765 - 82637) | 78591 (31617 - 161867) | 263 (89 - 641) | 419 (122 - 1094) |
| Seychelles | 549 (313 - 937) | 941 (376 - 1867) | 3 (1 - 8) | 5 (1 - 13) |
| Sierra Leone | 100493 (59156 - 166950) | 137490 (61834 - 251113) | 555 (195 - 1303) | 722 (220 - 1839) |
| Singapore | 26062 (13516 - 49057) | 18299 (1566 - 64247) | 146 (48 - 363) | 98 (6 - 396) |
| Slovakia | 28514 (14932 - 51531) | 50680 (21085 - 109855) | 159 (55 - 404) | 269 (80 - 695) |
| Slovenia | 6696 (3335 - 12810) | 11502 (3547 - 26046) | 36 (12 - 94) | 60 (13 - 165) |
| Solomon Islands | 3618 (2070 - 5813) | 6866 (3178 - 12522) | 21 (7 - 50) | 38 (11 - 96) |
| Somalia | 91595 (51625 - 150842) | 214543 (108124 - 381213) | 520 (179 - 1229) | 1169 (385 - 2849) |
| South Africa | 242768 (126429 - 459524) | 564552 (178120 - 1273505) | 1385 (457 - 3664) | 3038 (759 - 8289) |
| South Sudan | 36377 (20846 - 60407) | 100303 (50253 - 179055) | 206 (70 - 508) | 546 (168 - 1339) |
| Spain | 121961 (64490 - 210198) | 136108 (18766 - 358903) | 730 (250 - 1781) | 753 (76 - 2350) |
| Sri Lanka | 77942 (43370 - 127416) | 183588 (67464 - 366837) | 453 (164 - 1051) | 997 (261 - 2544) |
| Sudan | 517925 (307816 - 842838) | 757022 (396287 - 1294129) | 2974 (1067 - 6757) | 4086 (1371 - 9725) |
| Suriname | 4258 (2424 - 7170) | 8403 (4131 - 15027) | 25 (9 - 60) | 46 (15 - 121) |
| Sweden | 65518 (35592 - 112544) | 100556 (38852 - 194729) | 399 (145 - 975) | 565 (158 - 1543) |
| Switzerland | 36885 (19355 - 61912) | 49304 (13913 - 107443) | 226 (77 - 563) | 277 (62 - 797) |
| Syrian Arab Republic | 60211 (33060 - 97445) | 131615 (59933 - 235991) | 354 (122 - 843) | 725 (209 - 1806) |
| Taiwan (Province of China) | 143597 (79585 - 244418) | 369466 (173287 - 687719) | 776 (265 - 1937) | 1925 (576 - 4775) |
| Tajikistan | 30581 (22762 - 41372) | 33510 (21602 - 46127) | 187 (77 - 403) | 194 (72 - 443) |
| Thailand | 254055 (142485 - 417838) | 571872 (217570 - 1103625) | 1517 (543 - 3449) | 3169 (825 - 8056) |
| Timor-Leste | 4364 (2446 - 7104) | 8939 (4102 - 16409) | 26 (9 - 61) | 50 (15 - 120) |
| Togo | 69430 (40265 - 112459) | 80306 (28385 - 163791) | 398 (143 - 945) | 430 (110 - 1145) |
| Tokelau | 7 (4 - 11) | 13 (6 - 25) | 0 (0 - 0) | 0 (0 - 0) |
| Tonga | 454 (257 - 733) | 1010 (425 - 1854) | 3 (1 - 6) | 6 (2 - 14) |
| Trinidad and Tobago | 12776 (7336 - 21418) | 26027 (13452 - 46485) | 75 (28 - 174) | 143 (48 - 368) |
| Tunisia | 66607 (37557 - 107108) | 119052 (56105 - 220149) | 406 (144 - 949) | 670 (197 - 1660) |
| Turkey | 485685 (265578 - 803122) | 811791 (352581 - 1511078) | 2915 (1059 - 7002) | 4511 (1290 - 11630) |
| Turkmenistan | 30548 (16953 - 52691) | 46457 (20257 - 92568) | 176 (66 - 422) | 253 (76 - 643) |
| Tuvalu | 69 (39 - 112) | 119 (54 - 219) | 0 (0 - 1) | 1 (0 - 2) |
| Uganda | 63607 (45640 - 84992) | 108004 (70117 - 150324) | 353 (141 - 766) | 584 (208 - 1282) |
| Ukraine | 413531 (227763 - 695316) | 688457 (337132 - 1308650) | 2470 (871 - 6231) | 3872 (1321 - 10213) |
| United Arab Emirates | 132125 (72052 - 233643) | 87699 (38397 - 164375) | 757 (254 - 1885) | 473 (126 - 1165) |
| United Kingdom | 386351 (210553 - 646616) | 455080 (93421 - 1045815) | 2369 (852 - 5754) | 2572 (445 - 7815) |
| United Republic of Tanzania | 89711 (63775 - 123619) | 501885 (417743 - 597445) | 492 (181 - 1080) | 2671 (1080 - 5532) |
| United States of America | 1642004 (892648 - 2778226) | 1396795 (208173 - 3438035) | 10134 (3606 - 25250) | 8025 (976 - 25097) |
| United States Virgin Islands | 497 (280 - 822) | 948 (463 - 1759) | 3 (1 - 7) | 5 (2 - 13) |
| Uruguay | 16459 (9284 - 27879) | 18948 (4254 - 41382) | 98 (36 - 239) | 105 (18 - 310) |
| Uzbekistan | 156695 (88272 - 267432) | 256625 (98734 - 509274) | 904 (331 - 2072) | 1389 (407 - 3749) |
| Vanuatu | 1633 (942 - 2646) | 3164 (1491 - 5694) | 9 (3 - 23) | 17 (6 - 43) |
| Venezuela (Bolivarian Republic of) | 120597 (66056 - 207807) | 213844 (77431 - 440384) | 684 (244 - 1650) | 1152 (286 - 3090) |
| Viet Nam | 252964 (143423 - 443132) | 449459 (113365 - 985469) | 1450 (496 - 3463) | 2427 (475 - 6870) |
| Yemen | 248003 (143314 - 404762) | 428501 (231588 - 717519) | 1492 (536 - 3581) | 2409 (784 - 5939) |
| Zambia | 71386 (38720 - 120319) | 187200 (84708 - 350078) | 399 (141 - 945) | 1004 (315 - 2483) |
| Zimbabwe | 45590 (24456 - 81116) | 128921 (48224 - 269824) | 252 (83 - 610) | 684 (189 - 1747) |

DALYs, disability-adjusted life-years; UI, uncertainty interval

**Table S5** ASPR and ASDR for infertility in 204 countries in 2021

| **Location** | **ASPR/100,000 (95% UI)** | | **ASDR/100,000 (95% UI)** | |
| --- | --- | --- | --- | --- |
|  | **Male** | **Female** | **Male** | **Female** |
| Afghanistan | 585.09 (432.08 - 764.44) | 787.89 (505.09 - 1083.29) | 3.31 (1.34 - 7.32) | 4.25 (1.54 - 9.71) |
| Albania | 669.86 (491.43 - 917.98) | 975.38 (708.81 - 1270.08) | 4.02 (1.56 - 8.81) | 5.45 (2.05 - 11.09) |
| Algeria | 1013.95 (565.16 - 1647.28) | 1710.6 (738.66 - 3052.16) | 6.1 (2.14 - 14.11) | 9.54 (2.77 - 24.17) |
| American Samoa | 1085.2 (612.04 - 1747.87) | 2013.9 (832.55 - 3735.02) | 6.22 (2.24 - 14.82) | 10.95 (3.25 - 26.51) |
| Andorra | 824.6 (435.71 - 1363.3) | 1060.44 (276.89 - 2236.9) | 5.14 (1.76 - 12.73) | 6.03 (1.24 - 16.92) |
| Angola | 1800.68 (1017.65 - 2952.35) | 3816.96 (2002.37 - 6678.34) | 10.02 (3.61 - 24.45) | 20.24 (6.69 - 47.47) |
| Antigua and Barbuda | 1505.75 (851.09 - 2451.31) | 2887.06 (1477.76 - 5235.94) | 8.85 (3.18 - 21.32) | 15.98 (5.27 - 41.34) |
| Argentina | 1022.31 (583.47 - 1729.93) | 1053.24 (254.95 - 2334.95) | 6.07 (2.21 - 14.97) | 5.91 (1.01 - 16.83) |
| Armenia | 431.14 (302.62 - 600.38) | 495.95 (283 - 733.71) | 2.47 (0.96 - 5.36) | 2.68 (0.93 - 6.08) |
| Australia | 566.75 (318.15 - 969.69) | 123.31 (34.12 - 515.39) | 3.43 (1.26 - 8.41) | 0.71 (0.12 - 3.04) |
| Austria | 1622.47 (907.62 - 2710.6) | 2908.71 (1312.34 - 5314.31) | 9.75 (3.47 - 24.43) | 16.07 (5.02 - 40.76) |
| Azerbaijan | 1271.84 (709.26 - 2129.58) | 2234.32 (957.83 - 4399.13) | 7.54 (2.67 - 18.24) | 12.4 (4.04 - 32.29) |
| Bahamas | 1526.59 (873.9 - 2552.71) | 2776.93 (1395.96 - 5143.25) | 8.97 (3.19 - 21.85) | 15.37 (5.01 - 38.78) |
| Bahrain | 1104.98 (635.05 - 1815.74) | 1875.6 (873.87 - 3329.38) | 6.68 (2.36 - 15.72) | 10.49 (2.99 - 26.36) |
| Bangladesh | 741.06 (554.73 - 981.89) | 1550.85 (1224.08 - 1863.47) | 4.44 (1.78 - 9.44) | 8.86 (3.44 - 17.5) |
| Barbados | 1502.12 (851.23 - 2491.66) | 2851.21 (1450.79 - 5186.28) | 8.84 (3.13 - 21.62) | 15.76 (5.24 - 40.09) |
| Belarus | 1426.76 (798.91 - 2445.33) | 2565.89 (1176.63 - 4823.43) | 8.48 (2.96 - 21.36) | 14.29 (4.48 - 36.29) |
| Belgium | 1443.21 (797.5 - 2320.8) | 2498.25 (1108.32 - 4533.2) | 8.78 (3.07 - 21.68) | 13.94 (3.94 - 34.84) |
| Belize | 942.42 (534.24 - 1544.25) | 1403.46 (538.91 - 2718.62) | 5.69 (2.09 - 13.37) | 7.94 (2.24 - 19.9) |
| Benin | 1115.31 (826.91 - 1471.57) | 691.43 (262.8 - 1155.35) | 6.2 (2.46 - 13.5) | 3.69 (1.05 - 9.82) |
| Bermuda | 1503.38 (851.36 - 2490.79) | 2864.06 (1465.61 - 5206.64) | 8.86 (3.15 - 21.59) | 15.81 (5.34 - 41.26) |
| Bhutan | 699.68 (409.08 - 1141.15) | 1382.93 (581.38 - 2585.81) | 4.19 (1.45 - 9.87) | 7.88 (2.3 - 18.86) |
| Bolivia (Plurinational State of) | 617.82 (343.9 - 1046.34) | 387.39 (44.56 - 1262.32) | 3.48 (1.22 - 7.99) | 2.09 (0.16 - 8.01) |
| Bosnia and Herzegovina | 1028.5 (535.53 - 1769.41) | 1912.52 (796.38 - 3897.24) | 5.79 (1.96 - 14.61) | 10.24 (2.95 - 26.01) |
| Botswana | 793.34 (411.81 - 1460.55) | 2248.96 (894.14 - 4619.26) | 4.32 (1.48 - 10.68) | 11.71 (3.19 - 31.58) |
| Brazil | 1106.42 (626.91 - 1857.81) | 1677.47 (709.28 - 3278.73) | 6.52 (2.36 - 15.68) | 9.25 (2.94 - 25.12) |
| Brunei Darussalam | 658.79 (359.22 - 1143.25) | 347.96 (40.59 - 1240.4) | 3.8 (1.34 - 9.52) | 1.88 (0.14 - 7.97) |
| Bulgaria | 1050.82 (550.05 - 1830.74) | 1933.64 (797.8 - 3846.96) | 5.92 (2.02 - 14.92) | 10.32 (2.97 - 25.79) |
| Burkina Faso | 1637.74 (951.28 - 2682.6) | 1478.92 (429.12 - 3252.34) | 9.15 (3.27 - 21.8) | 7.86 (1.62 - 22.19) |
| Burundi | 286.34 (210.07 - 378.98) | 344 (191.58 - 521.41) | 1.61 (0.64 - 3.54) | 1.87 (0.65 - 4.66) |
| Cabo Verde | 2173.28 (1263.58 - 3422.38) | 2767.17 (1159.23 - 5283.78) | 12.13 (4.37 - 28.36) | 14.51 (4.27 - 35.47) |
| Cambodia | 969.59 (544.24 - 1676.42) | 2352.48 (1097.29 - 4398.01) | 5.39 (1.84 - 12.89) | 12.5 (3.89 - 31.23) |
| Cameroon | 3280.58 (1939.56 - 5141.3) | 3666.38 (1820.04 - 6492.82) | 18.96 (7.04 - 44.69) | 20.01 (6.4 - 51.75) |
| Canada | 633.56 (344.26 - 1052.91) | 472.43 (45.82 - 1256.53) | 3.98 (1.41 - 9.91) | 2.79 (0.22 - 9.13) |
| Central African Republic | 2803.4 (1618.38 - 4341.94) | 5751.56 (3585.62 - 9238.57) | 16 (5.92 - 37.42) | 31.4 (11.58 - 71.65) |
| Chad | 2129.96 (1188.81 - 3548.28) | 2112.24 (754.78 - 4255.64) | 11.49 (4.05 - 26.66) | 10.97 (2.73 - 28.2) |
| Chile | 997.51 (555.17 - 1695.3) | 1072.61 (244.32 - 2385.43) | 5.93 (2.17 - 14.78) | 5.95 (1.04 - 17.82) |
| China | 1591.79 (886.51 - 2708.22) | 4144.55 (2149.99 - 7228.3) | 8.66 (2.97 - 21.04) | 21.77 (7.48 - 54.69) |
| Colombia | 397.86 (299.24 - 531.64) | 213.19 (48.78 - 408.02) | 2.47 (0.99 - 5.18) | 1.26 (0.23 - 3.38) |
| Comoros | 1736.17 (970.5 - 2811.95) | 4663.64 (2603.13 - 7443.88) | 10.38 (3.69 - 24.65) | 26.39 (9.25 - 63.4) |
| Congo | 1387.03 (773.98 - 2332.26) | 3105.42 (1461.38 - 5756.32) | 7.55 (2.66 - 18.17) | 16.13 (4.75 - 41.73) |
| Cook Islands | 1035.22 (597.82 - 1686.39) | 2103.25 (936.96 - 3898.08) | 5.97 (2.15 - 14.07) | 11.45 (3.25 - 30.14) |
| Costa Rica | 1055.45 (590.51 - 1782.78) | 1791.2 (758.88 - 3586.31) | 6.06 (2.22 - 14.43) | 9.67 (2.68 - 26.4) |
| Coted'Ivoire | 2643.52 (1568.36 - 4217.07) | 3130.79 (1464.67 - 5887.37) | 14.62 (5.37 - 33.8) | 16.47 (4.91 - 41.66) |
| Croatia | 1055.15 (552.84 - 1818.32) | 1940.49 (779.27 - 4005.15) | 5.95 (2 - 14.83) | 10.39 (2.96 - 26.03) |
| Cuba | 1498.58 (851.33 - 2494.68) | 2894.76 (1502.44 - 5266.97) | 8.82 (3.24 - 21.03) | 15.94 (5.3 - 40.83) |
| Cyprus | 829.66 (440.59 - 1407.67) | 1104.12 (334.94 - 2355.03) | 5.16 (1.72 - 12.83) | 6.3 (1.45 - 17.03) |
| Czechia | 1409.93 (749.96 - 2463.73) | 2818.05 (1319.6 - 5200.36) | 7.87 (2.62 - 19.68) | 15.02 (4.67 - 38.38) |
| Democratic People's Republic of Korea | 1160.2 (643.73 - 2041.9) | 3035.53 (1493.44 - 5740.63) | 6.29 (2.12 - 15.17) | 15.9 (5 - 37.51) |
| Democratic Republic of the Congo | 1189.35 (661.73 - 1932.69) | 2239.74 (1020.35 - 4305.76) | 6.6 (2.22 - 16.11) | 11.9 (3.2 - 28.96) |
| Denmark | 450.55 (224.47 - 795.74) | 317.37 (27.29 - 889.46) | 2.97 (0.99 - 7.45) | 1.92 (0.13 - 6.37) |
| Djibouti | 1777.91 (1027.08 - 2864.62) | 5304.56 (3029.58 - 8632.08) | 9.95 (3.63 - 23.29) | 28.4 (10.08 - 70.49) |
| Dominica | 1519.64 (869.2 - 2519.88) | 2804.27 (1437.75 - 5220.3) | 8.93 (3.19 - 21.52) | 15.51 (5.2 - 39.14) |
| Dominican Republic | 1280.27 (731.51 - 2179.92) | 2384.15 (1080.53 - 4596.73) | 7.41 (2.68 - 18.14) | 13 (4.1 - 34.44) |
| Ecuador | 632.06 (351.73 - 1065.9) | 364.79 (46.01 - 1240.19) | 3.59 (1.27 - 8.73) | 1.97 (0.16 - 7.74) |
| Egypt | 833.05 (466.77 - 1349.87) | 1882.51 (847.29 - 3177.21) | 5.13 (1.78 - 11.89) | 10.81 (3.17 - 26.81) |
| El Salvador | 586.9 (321.39 - 1022.6) | 749.7 (121.74 - 1866.44) | 3.36 (1.18 - 8.05) | 4.04 (0.53 - 12.32) |
| Equatorial Guinea | 1752.89 (995.23 - 2833.08) | 3910.76 (2082.03 - 7019.98) | 9.8 (3.54 - 23.65) | 20.66 (6.89 - 49.61) |
| Eritrea | 1530.45 (858.31 - 2498.82) | 4420.25 (2431.49 - 7558.71) | 8.75 (3.18 - 20.67) | 24.09 (8.43 - 56.28) |
| Estonia | 1434.74 (783.6 - 2474.49) | 2614.71 (1215.59 - 4726.04) | 8.51 (2.96 - 21.12) | 14.5 (4.74 - 36.92) |
| Eswatini | 569.24 (299.16 - 1007.21) | 1247.95 (377.37 - 2664.4) | 3.27 (1.12 - 8.03) | 6.77 (1.59 - 19.37) |
| Ethiopia | 808.78 (451.22 - 1324.26) | 1900.9 (902.06 - 3501.64) | 4.56 (1.61 - 10.52) | 10.27 (3.33 - 25.68) |
| Fiji | 1505.91 (877.88 - 2366.25) | 3271.06 (1733.88 - 5607.08) | 8.86 (3.18 - 21.11) | 18.15 (5.99 - 43.85) |
| Finland | 536.25 (274.41 - 906.12) | 831.16 (201.13 - 1839.28) | 3.36 (1.07 - 8.17) | 4.76 (0.88 - 13.61) |
| France | 1182.59 (639.37 - 2003.82) | 2096.62 (860.26 - 3736.66) | 7.58 (2.55 - 18.26) | 12.38 (3.56 - 31.08) |
| Gabon | 2151.79 (1242.99 - 3432.98) | 5072.46 (2842.85 - 8423.78) | 12.11 (4.33 - 28.75) | 26.99 (9.09 - 67.98) |
| Gambia | 2223.41 (1321.41 - 3469.27) | 2487.04 (961.97 - 4995.8) | 12.45 (4.48 - 28.63) | 13.11 (3.62 - 34.08) |
| Georgia | 1039.13 (573.63 - 1803.86) | 1613.95 (616.59 - 3294.8) | 6 (2.2 - 14.39) | 8.81 (2.34 - 22.79) |
| Germany | 533.61 (280.06 - 944.23) | 563.52 (95.39 - 1435.02) | 3.33 (1.11 - 8.46) | 3.2 (0.41 - 10.34) |
| Ghana | 2264.66 (1305.25 - 3685.48) | 2103.66 (749.23 - 4600.61) | 12.62 (4.49 - 28.92) | 11.06 (2.58 - 27.76) |
| Greece | 828.79 (433.8 - 1420.68) | 1024.36 (241.8 - 2277.84) | 5.16 (1.72 - 12.99) | 5.78 (1.07 - 16.42) |
| Greenland | 638.6 (343.03 - 1056.92) | 476.15 (46.9 - 1269.26) | 4.01 (1.35 - 10.35) | 2.82 (0.24 - 9.62) |
| Grenada | 1546.13 (869.85 - 2566.14) | 2869.61 (1469.17 - 5286.55) | 9.05 (3.26 - 22.36) | 15.85 (5 - 40.67) |
| Guam | 1080.05 (616.2 - 1738.58) | 1973.44 (761.4 - 3710.92) | 6.22 (2.25 - 14.6) | 10.77 (3.01 - 27.21) |
| Guatemala | 947.41 (516.05 - 1619.18) | 1589.75 (605.02 - 3413.33) | 5.22 (1.84 - 12.23) | 8.37 (2.29 - 22.19) |
| Guinea | 2048.65 (1516.62 - 2683.14) | 2069.83 (1311.82 - 2938.9) | 11.41 (4.54 - 25.13) | 11.05 (4.04 - 25.35) |
| Guinea-Bissau | 2273.99 (1302.62 - 3689.93) | 2165.53 (774 - 4168.98) | 12.62 (4.57 - 28.92) | 11.52 (2.88 - 28.25) |
| Guyana | 2014.24 (1156.2 - 3260.91) | 3971.96 (2171.02 - 7008.92) | 11.64 (4.22 - 27.09) | 21.67 (7.23 - 55.25) |
| Haiti | 1016.85 (752.44 - 1324.3) | 1559.03 (1151.86 - 1952.69) | 5.79 (2.29 - 12.37) | 8.49 (3.27 - 18.35) |
| Honduras | 967.91 (525.44 - 1646.92) | 1728.59 (682.8 - 3452.58) | 5.4 (1.86 - 12.87) | 9.15 (2.6 - 25.19) |
| Hungary | 1044.86 (542.26 - 1825.88) | 1919.84 (802.68 - 3982.19) | 5.87 (2.01 - 14.91) | 10.28 (3.07 - 25.94) |
| Iceland | 834.57 (438.59 - 1399.57) | 1147.73 (323.91 - 2469.44) | 5.2 (1.76 - 12.77) | 6.5 (1.52 - 18.22) |
| India | 1516.62 (872.42 - 2491.07) | 3768.2 (2080.6 - 6423.08) | 8.9 (3.22 - 20.61) | 20.91 (7.53 - 50.64) |
| Indonesia | 1939.83 (1128.75 - 3179.61) | 4022.14 (2093 - 7179.39) | 11.2 (4.06 - 27.52) | 21.97 (7.52 - 53.3) |
| Iran (Islamic Republic of) | 1267.29 (668.74 - 2212.22) | 1642.98 (723.46 - 3057.68) | 8.37 (2.8 - 19.43) | 10.2 (3.06 - 27.83) |
| Iraq | 1112.42 (634.49 - 1802.09) | 1885.95 (908.16 - 3257.92) | 6.67 (2.38 - 15.06) | 10.53 (3.35 - 27.07) |
| Ireland | 818.9 (434.66 - 1391.97) | 1131.66 (320.05 - 2431.56) | 5.06 (1.7 - 12.3) | 6.37 (1.33 - 17.44) |
| Israel | 1217.99 (648.36 - 2068.65) | 870.09 (211.07 - 1972.42) | 7.48 (2.54 - 18.74) | 4.89 (0.92 - 14.43) |
| Italy | 1325.59 (731.69 - 2306.54) | 1085.93 (140.65 - 3057.65) | 8.04 (2.89 - 19.69) | 6.02 (0.67 - 21.41) |
| Jamaica | 1767.87 (1021.65 - 2865.6) | 3408.47 (1856.4 - 5920.5) | 10.36 (3.79 - 23.86) | 18.8 (6.29 - 46.66) |
| Japan | 1040.13 (565.18 - 1732.58) | 720.15 (71.71 - 2225.72) | 5.98 (2.09 - 14.97) | 3.85 (0.27 - 14.45) |
| Jordan | 944.05 (691.26 - 1287.07) | 1657.33 (1233.93 - 2111.54) | 5.74 (2.28 - 12.49) | 9.25 (3.53 - 20.47) |
| Kazakhstan | 1022.13 (566.92 - 1753.78) | 1739.32 (715.42 - 3543.33) | 5.96 (2.24 - 14.29) | 9.55 (2.76 - 23.72) |
| Kenya | 957.26 (537.89 - 1633.13) | 2457.26 (1216.07 - 4516.83) | 5.31 (1.83 - 13.11) | 13.07 (4.22 - 31.81) |
| Kiribati | 1064.75 (607.78 - 1723.37) | 2166.8 (985.98 - 4031.19) | 6.12 (2.2 - 14.42) | 11.81 (3.62 - 29.68) |
| Kuwait | 1119.75 (633.65 - 1808.15) | 1809.5 (837.2 - 3230.73) | 6.74 (2.42 - 15.56) | 10.1 (2.95 - 25.62) |
| Kyrgyzstan | 1251.82 (709.87 - 2121.36) | 2376.02 (1128.85 - 4490.12) | 7.16 (2.66 - 16.98) | 12.84 (4.27 - 31.74) |
| Lao People's Democratic Republic | 885.62 (496.59 - 1488.89) | 1965.78 (889.7 - 3746.2) | 5.12 (1.77 - 12.37) | 10.74 (3.14 - 27.62) |
| Latvia | 1458.48 (794.22 - 2510.5) | 2677.86 (1282.01 - 5050.99) | 8.53 (2.96 - 21.31) | 14.71 (4.74 - 37.8) |
| Lebanon | 1379.03 (808.81 - 2210.39) | 2690.64 (1381.6 - 4718.52) | 8.14 (2.9 - 19.24) | 14.71 (4.48 - 34.22) |
| Lesotho | 1025.55 (566.9 - 1750.15) | 2903.78 (1367.28 - 5463.55) | 5.81 (2.01 - 14.37) | 15.52 (4.87 - 40.08) |
| Liberia | 2804.1 (1612.32 - 4491.78) | 3279.87 (1503.51 - 6204.2) | 15.38 (5.58 - 36.61) | 17.07 (4.92 - 41.92) |
| Libya | 1022.64 (571.4 - 1625.81) | 1659.59 (739.84 - 3042.8) | 6.26 (2.24 - 14.7) | 9.39 (2.63 - 23.47) |
| Lithuania | 1461.14 (805.31 - 2563.38) | 2748.21 (1318.16 - 5247.49) | 8.42 (2.96 - 20.81) | 14.93 (4.88 - 38.56) |
| Luxembourg | 830.98 (442.67 - 1394.71) | 998.68 (241.54 - 2204.31) | 5.15 (1.8 - 13.34) | 5.66 (1.12 - 15.8) |
| Madagascar | 1042.23 (596.05 - 1699.28) | 2586.92 (1289.44 - 4409.9) | 6.1 (2.17 - 14.58) | 14.41 (4.75 - 35.41) |
| Malawi | 297.57 (209.93 - 401.32) | 391.45 (241.46 - 540.38) | 1.62 (0.65 - 3.49) | 2.1 (0.77 - 4.94) |
| Malaysia | 829.28 (461.2 - 1436.99) | 2325.36 (933.35 - 4620.9) | 4.63 (1.59 - 11.46) | 12.28 (3.46 - 31.97) |
| Maldives | 1459.44 (1109.85 - 1890.73) | 3476.5 (2747.9 - 4253.32) | 8.96 (3.59 - 19.27) | 19.79 (7.87 - 40.86) |
| Mali | 1476.27 (1112.9 - 1937.96) | 932.19 (504.72 - 1380.79) | 8.4 (3.24 - 17.65) | 5.17 (1.71 - 11.42) |
| Malta | 833.3 (438.53 - 1394.59) | 1146.15 (333.53 - 2427.94) | 5.17 (1.8 - 12.81) | 6.47 (1.51 - 17.37) |
| Marshall Islands | 1085.85 (624.85 - 1753.29) | 2151.06 (1006.16 - 3896.22) | 6.22 (2.22 - 14.97) | 11.74 (3.61 - 29.15) |
| Mauritania | 2707.04 (1625.71 - 4341.97) | 2889.64 (1229.34 - 5615.82) | 15.59 (5.6 - 37.2) | 15.75 (4.48 - 42.68) |
| Mauritius | 858.68 (472.23 - 1450.6) | 1911.4 (728.19 - 3753.42) | 4.95 (1.81 - 12.07) | 10.28 (2.91 - 27) |
| Mexico | 1384.45 (784.55 - 2354.71) | 2269.08 (968.71 - 4496.99) | 7.75 (2.77 - 18.06) | 12 (3.53 - 32.06) |
| Micronesia (Federated States of) | 1085.67 (634.42 - 1736.56) | 2131.27 (990.54 - 3912.83) | 6.24 (2.23 - 14.97) | 11.64 (3.55 - 29.08) |
| Monaco | 823.83 (440.46 - 1389.29) | 1070.87 (288.67 - 2229.92) | 5.13 (1.71 - 12.67) | 6.06 (1.3 - 17.11) |
| Mongolia | 1039.15 (585.92 - 1779.07) | 1731.64 (727.23 - 3392.88) | 5.98 (2.23 - 14.15) | 9.44 (2.89 - 24.57) |
| Montenegro | 1035.01 (546.97 - 1762.6) | 1851.44 (772.99 - 3869.42) | 5.84 (1.99 - 14.62) | 9.93 (2.87 - 25.34) |
| Morocco | 1322.62 (743.02 - 2155.35) | 2169.24 (1080.58 - 3696.51) | 8.13 (2.84 - 19.45) | 12.44 (3.77 - 30.71) |
| Mozambique | 1473.73 (830.14 - 2404.03) | 4159.35 (2301.08 - 6981.69) | 8.28 (3 - 19.87) | 22.12 (7.68 - 54.45) |
| Myanmar | 564.13 (421.63 - 743.11) | 998.72 (680.99 - 1305.84) | 3.34 (1.31 - 6.96) | 5.55 (2.1 - 12.53) |
| Namibia | 612 (327.93 - 1101.72) | 1501.89 (485.5 - 3295.1) | 3.48 (1.21 - 8.62) | 8.1 (2.01 - 22.3) |
| Nauru | 1056.65 (592.65 - 1720.19) | 2168.05 (968.26 - 4036.3) | 6.08 (2.2 - 14.39) | 11.78 (3.5 - 29.57) |
| Nepal | 475.37 (350.54 - 629.31) | 760.61 (553.04 - 985.18) | 2.94 (1.16 - 6.15) | 4.49 (1.75 - 9.44) |
| Netherlands | 821.89 (429.87 - 1394.52) | 1152.19 (334.13 - 2467.77) | 5.11 (1.76 - 12.64) | 6.55 (1.59 - 17.83) |
| New Zealand | 897.58 (497.25 - 1540.71) | 308.22 (52.6 - 1186.52) | 5.35 (1.91 - 13.08) | 1.72 (0.18 - 7.33) |
| Nicaragua | 646.76 (352.63 - 1114.98) | 889.79 (222.47 - 2023.12) | 3.72 (1.33 - 8.62) | 4.81 (0.88 - 14.65) |
| Niger | 2153.52 (1270.9 - 3422.11) | 2516.34 (1021.62 - 4808.68) | 12.11 (4.41 - 28.21) | 13.35 (3.61 - 32.4) |
| Nigeria | 1550.97 (890.03 - 2503.5) | 1813.15 (643.78 - 3910.41) | 8.72 (3.14 - 21.06) | 9.69 (2.27 - 25.34) |
| Niue | 1039.48 (583 - 1684.54) | 2137.52 (911.7 - 3918.44) | 5.99 (2.15 - 14.56) | 11.61 (3.39 - 29.21) |
| North Macedonia | 1028.77 (535.62 - 1776.31) | 1905.55 (809.03 - 3898.59) | 5.81 (2.05 - 14.28) | 10.2 (2.9 - 25.8) |
| Northern Mariana Islands | 1064.5 (607.57 - 1707.35) | 2098.88 (939.52 - 4015.36) | 6.13 (2.15 - 14.7) | 11.48 (3.41 - 28.07) |
| Norway | 1319.64 (719.51 - 2260.11) | 1803.04 (565.22 - 3639.91) | 8.15 (2.95 - 19.88) | 10.24 (2.46 - 27.37) |
| Oman | 1101.38 (634.23 - 1768.64) | 1935.46 (896.75 - 3348.43) | 6.64 (2.36 - 15.43) | 10.78 (3.07 - 25.63) |
| Pakistan | 1735.86 (602.15 - 3629.48) | 3877.12 (1093.75 - 8389.93) | 10.76 (2.74 - 31.09) | 22.81 (4.71 - 63.92) |
| Palau | 1037.84 (576.22 - 1660.38) | 2126.49 (908.22 - 3976.21) | 5.98 (2.14 - 13.93) | 11.55 (3.41 - 28.96) |
| Palestine | 1106.37 (639.04 - 1763.44) | 1956.53 (971.75 - 3352.1) | 6.63 (2.38 - 15.36) | 10.91 (3.2 - 26.77) |
| Panama | 1155.84 (644.44 - 1996.73) | 2140.89 (909.81 - 4237.6) | 6.41 (2.3 - 14.94) | 11.33 (3.37 - 29.51) |
| Papua New Guinea | 572.11 (415.45 - 779.24) | 685.44 (383.36 - 1029.27) | 3.26 (1.25 - 7.08) | 3.81 (1.23 - 9.45) |
| Paraguay | 1243.9 (692.19 - 1989.8) | 2371.86 (1280.85 - 4138.73) | 7.15 (2.59 - 16.52) | 12.97 (4.37 - 32.2) |
| Peru | 679.27 (368.53 - 1223.92) | 607.6 (53.27 - 1845.49) | 3.8 (1.3 - 9.17) | 3.25 (0.24 - 12.26) |
| Philippines | 1968.48 (817.08 - 3700.04) | 4277.68 (1477.29 - 8456.9) | 11.94 (3.53 - 32.06) | 24.49 (6.53 - 60.86) |
| Poland | 1921.05 (1065.25 - 3372.64) | 3493.73 (1745.33 - 6263.72) | 10.96 (3.82 - 27.81) | 18.92 (6.04 - 47.26) |
| Portugal | 779.16 (410.03 - 1327.82) | 1045.25 (290.93 - 2239.9) | 4.83 (1.65 - 12.06) | 5.93 (1.34 - 16.59) |
| Puerto Rico | 1503.71 (852.69 - 2473.6) | 2803.15 (1362.47 - 5060.97) | 8.85 (3.24 - 21.59) | 15.47 (5.11 - 39.66) |
| Qatar | 1102.31 (619.03 - 1776.68) | 1889.98 (819.06 - 3328.85) | 6.67 (2.37 - 15.44) | 10.53 (3.11 - 24.98) |
| Republic of Korea | 657.2 (355.47 - 1134.32) | 392.25 (40.69 - 1357.69) | 3.8 (1.32 - 9.44) | 2.12 (0.16 - 9.31) |
| Republic of Moldova | 1589.69 (889.67 - 2663.4) | 2749.66 (1337.88 - 4934.14) | 9.5 (3.47 - 23.83) | 15.42 (5.25 - 37.29) |
| Romania | 1063.13 (552.33 - 1886.81) | 1890.42 (782.1 - 3878.42) | 5.98 (2.05 - 14.97) | 10.11 (2.9 - 26.17) |
| Russian Federation | 2155.7 (1178.35 - 3631.64) | 3819.34 (1945.65 - 6906.25) | 12.69 (4.41 - 31.71) | 21.18 (7.51 - 53.81) |
| Rwanda | 801.84 (458.06 - 1355.73) | 668.32 (159.19 - 1579.44) | 4.58 (1.57 - 11.64) | 3.62 (0.64 - 10.05) |
| Saint Kitts and Nevis | 1519.1 (845.47 - 2533.08) | 2905.37 (1502.43 - 5248.84) | 8.92 (3.29 - 21.68) | 16.03 (5.2 - 41.13) |
| Saint Lucia | 1537.07 (857.59 - 2549.45) | 2887.65 (1454.91 - 5328.51) | 8.98 (3.25 - 22.19) | 15.92 (5.19 - 39.52) |
| Saint Vincent and the Grenadines | 1543.06 (862.78 - 2550.31) | 2862.37 (1444.34 - 5255.17) | 9.02 (3.31 - 22.18) | 15.79 (5.06 - 40.9) |
| Samoa | 1052.56 (607.36 - 1717) | 2152.41 (955.08 - 4066.51) | 6.05 (2.17 - 14.17) | 11.76 (3.71 - 29.59) |
| San Marino | 823.98 (433.85 - 1406.69) | 1090.02 (307.22 - 2447.61) | 5.13 (1.77 - 12.15) | 6.18 (1.22 - 17.37) |
| Sao Tome and Principe | 1262.67 (725.14 - 2068.36) | 1077.14 (243.36 - 2664.89) | 7.07 (2.52 - 16.74) | 5.72 (0.93 - 15.89) |
| Saudi Arabia | 1100.83 (634.34 - 1765.58) | 1913.01 (865.15 - 3317.9) | 6.63 (2.36 - 15.47) | 10.65 (3.11 - 26.54) |
| Senegal | 1704.89 (1300.51 - 2210.91) | 1594.39 (984.08 - 2276.63) | 9.82 (3.83 - 21.72) | 8.64 (3.09 - 19.31) |
| Serbia | 1030.32 (535.47 - 1769.06) | 1900.76 (784.82 - 3786.23) | 5.83 (1.95 - 14.57) | 10.18 (3 - 26.1) |
| Seychelles | 857 (484.56 - 1487.68) | 1874.9 (731.59 - 3699.17) | 4.96 (1.8 - 12.01) | 10.18 (2.75 - 25.89) |
| Sierra Leone | 2472.82 (1436 - 4038.42) | 3258.99 (1435.34 - 6064.16) | 13.5 (4.85 - 31.32) | 16.97 (4.97 - 43.2) |
| Singapore | 671.48 (365.23 - 1177.17) | 427.98 (42.71 - 1492.22) | 3.86 (1.33 - 9.96) | 2.3 (0.16 - 9.33) |
| Slovakia | 1037.08 (544.37 - 1824.02) | 1912.78 (812.85 - 3853.26) | 5.86 (2.02 - 14.84) | 10.25 (3.05 - 26.49) |
| Slovenia | 707.08 (345.78 - 1339.98) | 1323.93 (424.7 - 3017.45) | 3.83 (1.27 - 10.04) | 6.92 (1.65 - 19.63) |
| Solomon Islands | 1107.15 (634.02 - 1784.96) | 2087.25 (952.36 - 3871.2) | 6.35 (2.2 - 15.09) | 11.43 (3.52 - 29.14) |
| Somalia | 962.85 (544.73 - 1607.91) | 2400.6 (1169.54 - 4337.28) | 5.41 (1.9 - 12.94) | 12.95 (4.2 - 31.15) |
| South Africa | 722.59 (377.22 - 1360.38) | 1682.43 (544.6 - 3745.64) | 4.14 (1.39 - 10.87) | 9.08 (2.28 - 24.46) |
| South Sudan | 990.59 (556.33 - 1667.32) | 2336.03 (1142.77 - 4264.51) | 5.55 (1.92 - 13.38) | 12.65 (3.82 - 31.44) |
| Spain | 561.47 (300.35 - 972.52) | 572.24 (81.55 - 1489.18) | 3.43 (1.19 - 8.64) | 3.2 (0.31 - 10.4) |
| Sri Lanka | 722.34 (406.77 - 1204.21) | 1571.58 (581.82 - 3135.72) | 4.21 (1.52 - 9.85) | 8.56 (2.25 - 21.56) |
| Sudan | 2455.48 (1457.2 - 3947.53) | 3450.95 (1816.79 - 5950.05) | 13.94 (5.03 - 32.47) | 18.52 (6.1 - 44.27) |
| Suriname | 1511.69 (857.56 - 2537.77) | 2858.48 (1408.71 - 5123.09) | 8.87 (3.2 - 21.42) | 15.73 (5.1 - 41.25) |
| Sweden | 1343.91 (725.39 - 2286.74) | 2132.36 (823.32 - 4120.89) | 8.26 (2.97 - 20.07) | 12.08 (3.37 - 32.53) |
| Switzerland | 826.04 (438.6 - 1389.73) | 1093.2 (300.42 - 2460.85) | 5.13 (1.75 - 12.99) | 6.2 (1.37 - 17.52) |
| Syrian Arab Republic | 1024.34 (582.8 - 1652.28) | 1741.11 (800.69 - 3050.58) | 6.05 (2.14 - 13.92) | 9.59 (2.73 - 22.82) |
| Taiwan (Province of China) | 1139.03 (631.81 - 1946.01) | 2914.21 (1391.86 - 5524.41) | 6.19 (2.13 - 15.36) | 15.23 (4.75 - 38.92) |
| Tajikistan | 554.83 (415.53 - 750.94) | 594.41 (378.97 - 824.79) | 3.39 (1.39 - 7.28) | 3.45 (1.27 - 7.89) |
| Thailand | 807.69 (442.67 - 1314.54) | 1679.8 (619.89 - 3243.32) | 4.86 (1.72 - 11.22) | 9.35 (2.37 - 23.84) |
| Timor-Leste | 723.28 (408.05 - 1174.29) | 1504.43 (685.48 - 2778.19) | 4.22 (1.51 - 10.38) | 8.32 (2.53 - 20.9) |
| Togo | 1854.41 (1088.48 - 3038.52) | 1887.91 (660.7 - 3866.92) | 10.5 (3.81 - 25.14) | 10.07 (2.43 - 27.24) |
| Tokelau | 1050.32 (596.57 - 1706.06) | 2104.7 (890.11 - 3922.48) | 6.05 (2.17 - 14.28) | 11.45 (3.45 - 29.66) |
| Tonga | 1039.51 (587.05 - 1694.6) | 2110.18 (890.92 - 3912.36) | 5.99 (2.17 - 13.95) | 11.48 (3.37 - 28.96) |
| Trinidad and Tobago | 1733.57 (974.88 - 2846.91) | 3519 (1904.79 - 6203.92) | 10.28 (3.73 - 23.92) | 19.44 (6.45 - 49.06) |
| Tunisia | 1083.82 (605.57 - 1753.84) | 1799.92 (882.22 - 3163.78) | 6.68 (2.33 - 15.82) | 10.23 (2.98 - 25.39) |
| Turkey | 1062 (582.08 - 1734.84) | 1802.25 (763.44 - 3340.34) | 6.4 (2.33 - 15.62) | 10.05 (2.9 - 25.74) |
| Turkmenistan | 1040.96 (577.09 - 1805.05) | 1822.09 (789.86 - 3610.71) | 6 (2.23 - 14.37) | 9.9 (2.96 - 25.2) |
| Tuvalu | 1052.97 (594.71 - 1696.2) | 2152.54 (946.95 - 4021.73) | 6.07 (2.18 - 14.03) | 11.71 (3.5 - 29.91) |
| Uganda | 358.47 (256.78 - 476.16) | 504.28 (326.79 - 713.46) | 1.97 (0.77 - 4.11) | 2.71 (0.97 - 5.97) |
| Ukraine | 1995.24 (1068.39 - 3407.16) | 3299.99 (1675.76 - 6039.66) | 12.14 (4.08 - 29.99) | 18.86 (6.43 - 47.35) |
| United Arab Emirates | 1098.5 (628.34 - 1777.85) | 1947.87 (897.98 - 3415.41) | 6.62 (2.36 - 15.04) | 10.79 (3.11 - 26.25) |
| United Kingdom | 1236.77 (666.7 - 2167.36) | 1371.3 (294.78 - 3111.88) | 7.64 (2.74 - 18.61) | 7.8 (1.39 - 23.32) |
| United Republic of Tanzania | 365.37 (262.73 - 501.34) | 1800.95 (1504.07 - 2129.63) | 2 (0.74 - 4.42) | 9.54 (3.91 - 19.77) |
| United States of America | 1052.16 (569.1 - 1779.05) | 891.34 (134.53 - 2205.42) | 6.52 (2.29 - 16.4) | 5.14 (0.62 - 15.92) |
| United States Virgin Islands | 1518.59 (868.61 - 2547.66) | 2711.73 (1336.3 - 5024.13) | 8.93 (3.2 - 21.9) | 15.01 (4.85 - 38) |
| Uruguay | 998.8 (558.52 - 1689.03) | 1091 (241.74 - 2367.85) | 5.94 (2.17 - 14.53) | 6.08 (1.09 - 18.21) |
| Uzbekistan | 830.77 (471.44 - 1400.43) | 1350.33 (524.96 - 2682.56) | 4.8 (1.78 - 11.05) | 7.33 (2.15 - 19.58) |
| Vanuatu | 1105.65 (635.57 - 1791.4) | 2109.66 (986.6 - 3831.21) | 6.33 (2.23 - 15.2) | 11.56 (3.67 - 28.57) |
| Venezuela (Bolivarian Republic of) | 950.2 (522.03 - 1610.2) | 1477.17 (552.25 - 3058.07) | 5.44 (1.95 - 13.06) | 8 (2.06 - 22.05) |
| Viet Nam | 442.4 (249.91 - 761.02) | 790.28 (206.68 - 1710.79) | 2.55 (0.89 - 6.1) | 4.28 (0.85 - 11.96) |
| Yemen | 1505.89 (864.64 - 2416.56) | 2609.66 (1442.86 - 4376.39) | 9 (3.27 - 21.6) | 14.59 (4.72 - 36.52) |
| Zambia | 798.09 (433.91 - 1346.21) | 2031.27 (912.4 - 3833.97) | 4.43 (1.57 - 10.76) | 10.8 (3.26 - 27.31) |
| Zimbabwe | 660.95 (356.11 - 1208.12) | 1645.97 (604.65 - 3530.37) | 3.64 (1.22 - 8.89) | 8.69 (2.32 - 21.7) |

ASPR, age-standardized prevalence rate; ASDR, age-standardized disability-adjusted life-years rate; UI, uncertainty interval

**Table S6** EAPC of ASPR and ASDR for infertility in 204 countries and territories from 1990 to 2021

| **Location** | **EAPC of ASIR (95% CI)** | | **EAPC of ASDR (95% CI)** | |
| --- | --- | --- | --- | --- |
|  | **Male** | **Female** | **Male** | **Female** |
| Afghanistan | -1.39 (-1.65 to -1.13) | -2.5 (-2.93 to -2.07) | -1.23 (-1.47 to -1) | -2.38 (-2.79 to -1.98) |
| Albania | -1.18 (-1.86 to -0.49) | -1.48 (-2.56 to -0.39) | -1.08 (-1.73 to -0.44) | -1.43 (-2.47 to -0.38) |
| Algeria | 1.78 (1.55 to 2.02) | 2.87 (2.53 to 3.21) | 1.7 (1.44 to 1.96) | 2.75 (2.41 to 3.09) |
| American Samoa | -0.03 (-0.04 to -0.02) | -0.12 (-0.15 to -0.09) | -0.03 (-0.03 to -0.02) | -0.13 (-0.16 to -0.1) |
| Andorra | -0.04 (-0.07 to -0.02) | -0.23 (-0.29 to -0.16) | -0.04 (-0.06 to -0.02) | -0.24 (-0.3 to -0.17) |
| Angola | 0.01 (-0.02 to 0.04) | 0.24 (0.22 to 0.27) | 0.02 (0 to 0.05) | 0.26 (0.23 to 0.29) |
| Antigua and Barbuda | -0.01 (-0.02 to 0) | 0.05 (0.03 to 0.07) | 0 (-0.01 to 0.01) | 0.04 (0.02 to 0.06) |
| Argentina | -0.03 (-0.1 to 0.05) | -0.29 (-0.37 to -0.2) | -0.02 (-0.08 to 0.05) | -0.29 (-0.37 to -0.21) |
| Armenia | -1.93 (-2.24 to -1.61) | -2.7 (-3.29 to -2.1) | -1.95 (-2.24 to -1.67) | -2.76 (-3.32 to -2.2) |
| Australia | -0.06 (-0.11 to -0.01) | -0.19 (-0.36 to -0.02) | -0.04 (-0.09 to 0.01) | -0.19 (-0.37 to -0.02) |
| Austria | 0.75 (0.47 to 1.03) | 1.06 (0.74 to 1.38) | 0.78 (0.49 to 1.08) | 1.14 (0.78 to 1.49) |
| Azerbaijan | -0.39 (-1.21 to 0.43) | -0.39 (-1.64 to 0.87) | -0.35 (-1.1 to 0.4) | -0.38 (-1.57 to 0.82) |
| Bahamas | -0.04 (-0.05 to -0.03) | 0.06 (0.05 to 0.08) | -0.03 (-0.04 to -0.02) | 0.06 (0.04 to 0.07) |
| Bahrain | 0.01 (0 to 0.01) | 0.06 (0.03 to 0.09) | 0.01 (0.01 to 0.02) | 0.06 (0.03 to 0.09) |
| Bangladesh | 0.85 (0.53 to 1.16) | 2.96 (2.56 to 3.36) | 0.79 (0.5 to 1.07) | 2.78 (2.41 to 3.16) |
| Barbados | 0 (-0.02 to 0.01) | 0.02 (0 to 0.04) | 0 (-0.02 to 0.01) | 0.02 (0 to 0.04) |
| Belarus | -0.01 (-0.02 to -0.01) | 0.26 (0.22 to 0.3) | -0.01 (-0.02 to 0) | 0.23 (0.19 to 0.27) |
| Belgium | 1.54 (1.12 to 1.97) | 2.42 (1.89 to 2.95) | 1.48 (1.07 to 1.89) | 2.35 (1.83 to 2.87) |
| Belize | 0.99 (0.72 to 1.27) | 1.65 (1.21 to 2.09) | 0.89 (0.64 to 1.14) | 1.54 (1.13 to 1.95) |
| Benin | -1.27 (-1.33 to -1.2) | -1.88 (-2.15 to -1.6) | -1.25 (-1.32 to -1.18) | -1.84 (-2.1 to -1.58) |
| Bermuda | 0 (-0.01 to 0.01) | 0.07 (0.05 to 0.08) | 0 (0 to 0.01) | 0.06 (0.05 to 0.08) |
| Bhutan | -0.1 (-0.12 to -0.09) | -0.02 (-0.04 to 0) | -0.09 (-0.1 to -0.07) | -0.01 (-0.03 to 0) |
| Bolivia (Plurinational State of) | 1.39 (0.71 to 2.07) | 6.7 (3.89 to 9.57) | 1.34 (0.69 to 2) | 6.65 (3.88 to 9.5) |
| Bosnia and Herzegovina | -0.05 (-0.06 to -0.04) | 0.16 (0.13 to 0.2) | -0.04 (-0.05 to -0.02) | 0.15 (0.12 to 0.19) |
| Botswana | 0.48 (0.15 to 0.81) | 0.77 (0.26 to 1.29) | 0.48 (0.16 to 0.81) | 0.75 (0.25 to 1.26) |
| Brazil | 1.84 (1.41 to 2.28) | 1.7 (1.09 to 2.32) | 1.77 (1.34 to 2.2) | 1.62 (1 to 2.24) |
| Brunei Darussalam | -0.09 (-0.18 to 0) | -0.46 (-0.73 to -0.19) | -0.08 (-0.16 to 0) | -0.48 (-0.75 to -0.22) |
| Bulgaria | -0.01 (-0.02 to 0) | 0.03 (0.01 to 0.06) | 0 (-0.02 to 0.01) | 0.03 (0.01 to 0.06) |
| Burkina Faso | -0.73 (-1.24 to -0.22) | 2.15 (1.3 to 3.01) | -0.79 (-1.31 to -0.26) | 2.06 (1.21 to 2.91) |
| Burundi | -2.34 (-2.78 to -1.9) | -4.49 (-5.63 to -3.33) | -2.33 (-2.71 to -1.94) | -4.42 (-5.51 to -3.33) |
| Cabo Verde | -0.03 (-0.04 to -0.02) | 0.46 (0.41 to 0.51) | -0.03 (-0.04 to -0.02) | 0.43 (0.38 to 0.48) |
| Cambodia | -1.75 (-2.42 to -1.07) | -2.47 (-3.42 to -1.52) | -1.54 (-2.17 to -0.91) | -2.32 (-3.23 to -1.41) |
| Cameroon | -0.46 (-1.27 to 0.35) | 0.32 (-0.87 to 1.53) | -0.41 (-1.23 to 0.42) | 0.37 (-0.83 to 1.58) |
| Canada | -0.03 (-0.05 to 0) | -0.19 (-0.24 to -0.15) | -0.03 (-0.05 to 0) | -0.21 (-0.26 to -0.16) |
| Central African Republic | 0.72 (0.52 to 0.92) | 0.7 (0.47 to 0.93) | 0.73 (0.54 to 0.93) | 0.74 (0.51 to 0.96) |
| Chad | -2.63 (-3.31 to -1.94) | -4.22 (-5.49 to -2.93) | -2.64 (-3.32 to -1.95) | -4.14 (-5.38 to -2.87) |
| Chile | -0.1 (-0.17 to -0.03) | -0.17 (-0.29 to -0.04) | -0.09 (-0.15 to -0.03) | -0.21 (-0.33 to -0.08) |
| China | 0.01 (-0.05 to 0.06) | 0.01 (-0.03 to 0.05) | 0.03 (-0.04 to 0.09) | 0.02 (-0.03 to 0.07) |
| Colombia | 0.29 (0.12 to 0.47) | 1.23 (0.59 to 1.87) | 0.34 (0.2 to 0.49) | 1.35 (0.72 to 1.98) |
| Comoros | 0.89 (0.39 to 1.39) | 1.02 (0.46 to 1.58) | 1.03 (0.51 to 1.56) | 1.14 (0.56 to 1.73) |
| Congo | -0.63 (-1.15 to -0.11) | -0.52 (-1.27 to 0.25) | -0.66 (-1.17 to -0.14) | -0.54 (-1.29 to 0.22) |
| Cook Islands | 0 (-0.02 to 0.02) | -0.14 (-0.16 to -0.12) | 0 (-0.02 to 0.01) | -0.15 (-0.17 to -0.13) |
| Costa Rica | -0.04 (-0.05 to -0.04) | -0.06 (-0.08 to -0.04) | -0.04 (-0.05 to -0.04) | -0.08 (-0.1 to -0.05) |
| Coted'Ivoire | 0.81 (0.38 to 1.23) | 2.59 (1.87 to 3.33) | 0.81 (0.39 to 1.24) | 2.6 (1.88 to 3.32) |
| Croatia | -0.01 (-0.02 to -0.01) | -0.15 (-0.24 to -0.06) | -0.01 (-0.01 to 0) | -0.14 (-0.23 to -0.06) |
| Cuba | 0.01 (0 to 0.03) | -0.07 (-0.14 to 0.01) | 0.02 (0 to 0.03) | -0.06 (-0.12 to 0.01) |
| Cyprus | -0.08 (-0.11 to -0.06) | -0.49 (-0.66 to -0.31) | -0.07 (-0.09 to -0.05) | -0.51 (-0.68 to -0.34) |
| Czechia | 1.11 (0.69 to 1.52) | 1.44 (0.91 to 1.98) | 1.03 (0.64 to 1.42) | 1.4 (0.89 to 1.92) |
| Democratic People's Republic of Korea | 0.04 (0.01 to 0.06) | -0.05 (-0.08 to -0.02) | 0.04 (0.01 to 0.06) | -0.04 (-0.07 to -0.01) |
| Democratic Republic of the Congo | -0.76 (-1.59 to 0.09) | -0.8 (-2.35 to 0.78) | -0.72 (-1.52 to 0.09) | -0.73 (-2.26 to 0.81) |
| Denmark | 1.48 (1.03 to 1.92) | 3.66 (2.53 to 4.81) | 1.47 (1.03 to 1.91) | 3.63 (2.5 to 4.77) |
| Djibouti | 0.28 (-0.18 to 0.75) | 0.39 (-0.18 to 0.95) | 0.3 (-0.17 to 0.76) | 0.39 (-0.17 to 0.96) |
| Dominica | 0.01 (0 to 0.02) | 0.02 (0.01 to 0.03) | 0.01 (0 to 0.02) | 0.01 (0 to 0.03) |
| Dominican Republic | 1.34 (0.61 to 2.07) | 1.98 (0.76 to 3.22) | 1.26 (0.58 to 1.95) | 1.89 (0.71 to 3.08) |
| Ecuador | 2.18 (1.64 to 2.73) | 9.33 (7.27 to 11.42) | 2.07 (1.56 to 2.57) | 9.15 (7.13 to 11.2) |
| Egypt | 0.92 (0.58 to 1.26) | 1.36 (0.73 to 2) | 0.72 (0.4 to 1.05) | 1.13 (0.54 to 1.74) |
| El Salvador | 1.95 (1.17 to 2.74) | 5.16 (2.94 to 7.42) | 1.87 (1.1 to 2.64) | 5.07 (2.87 to 7.3) |
| Equatorial Guinea | -0.16 (-0.18 to -0.15) | 0.27 (0.22 to 0.33) | -0.11 (-0.13 to -0.1) | 0.28 (0.22 to 0.33) |
| Eritrea | 1.64 (1.45 to 1.83) | 2.27 (2.02 to 2.52) | 1.59 (1.42 to 1.77) | 2.18 (1.94 to 2.42) |
| Estonia | 0.01 (0 to 0.02) | 0.34 (0.29 to 0.38) | 0.01 (0 to 0.01) | 0.3 (0.26 to 0.34) |
| Eswatini | -0.39 (-1.12 to 0.34) | -0.64 (-2.39 to 1.13) | -0.41 (-1.13 to 0.32) | -0.68 (-2.4 to 1.08) |
| Ethiopia | -1.07 (-1.27 to -0.88) | -0.56 (-0.89 to -0.24) | -1.09 (-1.29 to -0.89) | -0.58 (-0.9 to -0.25) |
| Fiji | 0.02 (0.01 to 0.04) | -0.06 (-0.08 to -0.04) | 0.02 (0.01 to 0.03) | -0.07 (-0.09 to -0.05) |
| Finland | 1.05 (0.58 to 1.51) | 1.3 (0.52 to 2.09) | 1.05 (0.59 to 1.51) | 1.29 (0.52 to 2.07) |
| France | 1.57 (1.11 to 2.03) | 2.05 (1.46 to 2.64) | 1.5 (1.06 to 1.95) | 1.94 (1.38 to 2.51) |
| Gabon | 0.14 (-0.25 to 0.52) | 0.28 (-0.2 to 0.76) | 0.19 (-0.19 to 0.58) | 0.32 (-0.16 to 0.81) |
| Gambia | -0.52 (-0.82 to -0.21) | -0.57 (-1.01 to -0.13) | -0.51 (-0.8 to -0.21) | -0.56 (-0.99 to -0.13) |
| Georgia | -0.03 (-0.04 to -0.02) | -0.34 (-0.43 to -0.25) | -0.03 (-0.04 to -0.02) | -0.35 (-0.44 to -0.26) |
| Germany | 1.59 (1.14 to 2.04) | 4.7 (3.32 to 6.09) | 1.44 (1.03 to 1.86) | 4.48 (3.16 to 5.82) |
| Ghana | -0.16 (-0.64 to 0.32) | 0.4 (-0.48 to 1.29) | -0.18 (-0.67 to 0.3) | 0.38 (-0.5 to 1.27) |
| Greece | -0.04 (-0.07 to -0.01) | -0.39 (-0.51 to -0.26) | -0.05 (-0.07 to -0.02) | -0.42 (-0.54 to -0.31) |
| Greenland | -0.09 (-0.13 to -0.06) | -0.15 (-0.19 to -0.1) | -0.06 (-0.09 to -0.03) | -0.15 (-0.2 to -0.1) |
| Grenada | 0.01 (-0.01 to 0.03) | 0.03 (0.01 to 0.05) | 0.02 (0 to 0.03) | 0.03 (0.01 to 0.05) |
| Guam | 0.05 (0.04 to 0.07) | -0.28 (-0.32 to -0.25) | 0.05 (0.03 to 0.06) | -0.28 (-0.31 to -0.25) |
| Guatemala | -0.47 (-1.13 to 0.2) | -1.27 (-2.44 to -0.08) | -0.36 (-1 to 0.28) | -1.13 (-2.28 to 0.03) |
| Guinea | -0.79 (-0.9 to -0.69) | -0.2 (-0.51 to 0.11) | -0.77 (-0.86 to -0.68) | -0.16 (-0.46 to 0.13) |
| Guinea-Bissau | -0.01 (-0.02 to 0) | 1.01 (0.9 to 1.12) | 0 (-0.02 to 0.01) | 1 (0.9 to 1.11) |
| Guyana | -0.35 (-1.06 to 0.36) | -0.43 (-1.46 to 0.62) | -0.32 (-0.96 to 0.32) | -0.41 (-1.38 to 0.58) |
| Haiti | -0.41 (-0.57 to -0.25) | -0.19 (-0.4 to 0.02) | -0.47 (-0.63 to -0.3) | -0.24 (-0.44 to -0.04) |
| Honduras | 0.3 (-0.71 to 1.32) | 0.56 (-1.54 to 2.71) | 0.28 (-0.68 to 1.25) | 0.57 (-1.48 to 2.66) |
| Hungary | 0.01 (-0.01 to 0.02) | 0.01 (-0.02 to 0.05) | 0.01 (-0.01 to 0.02) | 0.02 (-0.02 to 0.06) |
| Iceland | -0.03 (-0.06 to -0.01) | -0.12 (-0.24 to 0.01) | -0.02 (-0.04 to 0) | -0.15 (-0.27 to -0.03) |
| India | 2.03 (1.54 to 2.53) | 2.53 (1.85 to 3.2) | 1.91 (1.45 to 2.37) | 2.39 (1.76 to 3.03) |
| Indonesia | 1.43 (0.98 to 1.88) | 1.67 (1.09 to 2.25) | 1.32 (0.9 to 1.74) | 1.58 (1.03 to 2.14) |
| Iran (Islamic Republic of) | -0.14 (-0.64 to 0.35) | -0.77 (-1.64 to 0.11) | -0.07 (-0.58 to 0.43) | -0.68 (-1.55 to 0.19) |
| Iraq | 0 (-0.01 to 0.02) | 0.12 (0.1 to 0.14) | 0.02 (0.01 to 0.04) | 0.11 (0.09 to 0.13) |
| Ireland | -0.06 (-0.1 to -0.03) | -0.05 (-0.14 to 0.04) | -0.07 (-0.09 to -0.04) | -0.09 (-0.18 to -0.01) |
| Israel | 0.07 (-0.03 to 0.16) | 0.28 (0.07 to 0.5) | 0.08 (-0.01 to 0.18) | 0.26 (0.04 to 0.48) |
| Italy | 1.39 (1.11 to 1.66) | 2.78 (2.34 to 3.23) | 1.45 (1.17 to 1.73) | 2.81 (2.36 to 3.26) |
| Jamaica | 0.02 (0 to 0.03) | 0.03 (0.01 to 0.06) | 0.02 (0.01 to 0.03) | 0.02 (0 to 0.04) |
| Japan | -0.11 (-0.2 to -0.02) | -0.36 (-0.65 to -0.06) | -0.11 (-0.19 to -0.02) | -0.36 (-0.65 to -0.08) |
| Jordan | 1.13 (0.9 to 1.37) | 2.33 (1.94 to 2.73) | 0.94 (0.69 to 1.19) | 2.1 (1.72 to 2.49) |
| Kazakhstan | 1.79 (1.38 to 2.21) | 3.27 (2.55 to 3.99) | 1.68 (1.28 to 2.09) | 3.13 (2.43 to 3.83) |
| Kenya | -2.59 (-3.73 to -1.42) | -3.88 (-5.58 to -2.14) | -2.58 (-3.73 to -1.42) | -3.89 (-5.6 to -2.14) |
| Kiribati | -0.02 (-0.03 to -0.01) | -0.05 (-0.08 to -0.02) | 0 (-0.01 to 0.01) | -0.05 (-0.07 to -0.02) |
| Kuwait | 0.02 (0.02 to 0.03) | -0.04 (-0.08 to -0.01) | 0.01 (0.01 to 0.02) | -0.07 (-0.11 to -0.03) |
| Kyrgyzstan | 0.3 (-0.11 to 0.72) | 0.63 (0.02 to 1.25) | 0.33 (-0.06 to 0.72) | 0.63 (0.04 to 1.22) |
| Lao People's Democratic Republic | 0.01 (-0.01 to 0.03) | -0.19 (-0.21 to -0.17) | 0.02 (0 to 0.04) | -0.19 (-0.21 to -0.17) |
| Latvia | 1.79 (1.44 to 2.13) | 2.72 (2.23 to 3.21) | 1.71 (1.39 to 2.04) | 2.62 (2.15 to 3.1) |
| Lebanon | 1.35 (0.88 to 1.82) | 1.94 (1.32 to 2.57) | 1.36 (0.89 to 1.83) | 1.92 (1.29 to 2.55) |
| Lesotho | -2.24 (-3.27 to -1.2) | -3.85 (-5.52 to -2.15) | -2.2 (-3.2 to -1.19) | -3.79 (-5.42 to -2.14) |
| Liberia | -0.66 (-1.14 to -0.17) | 0.07 (-0.52 to 0.66) | -0.71 (-1.22 to -0.19) | 0.02 (-0.59 to 0.63) |
| Libya | 1.48 (1.2 to 1.77) | 2.41 (1.95 to 2.87) | 1.46 (1.18 to 1.74) | 2.36 (1.9 to 2.81) |
| Lithuania | 1.73 (1.4 to 2.06) | 2.37 (1.93 to 2.82) | 1.64 (1.33 to 1.96) | 2.29 (1.87 to 2.72) |
| Luxembourg | -0.08 (-0.11 to -0.05) | -0.04 (-0.22 to 0.15) | -0.07 (-0.09 to -0.04) | -0.07 (-0.25 to 0.11) |
| Madagascar | 0.76 (0.14 to 1.37) | 1.27 (0.41 to 2.14) | 0.8 (0.23 to 1.38) | 1.31 (0.48 to 2.14) |
| Malawi | -4.22 (-4.55 to -3.9) | -6.2 (-6.65 to -5.75) | -4.31 (-4.64 to -3.99) | -6.18 (-6.62 to -5.73) |
| Malaysia | 0.2 (0.13 to 0.27) | -0.27 (-0.3 to -0.24) | 0.22 (0.15 to 0.3) | -0.28 (-0.31 to -0.25) |
| Maldives | -1.14 (-1.58 to -0.69) | -1.68 (-2.23 to -1.13) | -0.95 (-1.39 to -0.52) | -1.54 (-2.09 to -0.99) |
| Mali | -1.33 (-1.67 to -0.99) | 0.69 (-0.33 to 1.73) | -1.26 (-1.62 to -0.91) | 0.74 (-0.26 to 1.76) |
| Malta | -0.05 (-0.08 to -0.02) | -0.23 (-0.45 to -0.01) | -0.05 (-0.08 to -0.03) | -0.27 (-0.49 to -0.05) |
| Marshall Islands | 0 (-0.01 to 0.01) | -0.12 (-0.14 to -0.1) | 0.01 (0 to 0.02) | -0.12 (-0.15 to -0.1) |
| Mauritania | 1.66 (1.47 to 1.85) | 4.36 (3.86 to 4.86) | 1.66 (1.46 to 1.85) | 4.27 (3.77 to 4.76) |
| Mauritius | -0.05 (-0.06 to -0.04) | -0.25 (-0.27 to -0.22) | -0.05 (-0.06 to -0.04) | -0.26 (-0.29 to -0.24) |
| Mexico | 0.69 (0.29 to 1.1) | 1.49 (0.83 to 2.15) | 0.66 (0.27 to 1.06) | 1.47 (0.82 to 2.12) |
| Micronesia (Federated States of) | -0.05 (-0.06 to -0.04) | -0.01 (-0.03 to 0.01) | -0.03 (-0.04 to -0.02) | -0.01 (-0.03 to 0.01) |
| Monaco | -0.07 (-0.1 to -0.05) | -0.12 (-0.17 to -0.06) | -0.06 (-0.08 to -0.04) | -0.13 (-0.18 to -0.08) |
| Mongolia | 0.27 (0.2 to 0.34) | 0.34 (0.29 to 0.4) | 0.29 (0.21 to 0.37) | 0.36 (0.3 to 0.41) |
| Montenegro | -0.02 (-0.03 to -0.01) | -0.01 (-0.02 to 0.01) | -0.01 (-0.02 to 0) | -0.01 (-0.03 to 0.01) |
| Morocco | 2.79 (1.97 to 3.61) | 3.88 (2.68 to 5.09) | 2.74 (1.92 to 3.57) | 3.85 (2.64 to 5.06) |
| Mozambique | -1.19 (-1.8 to -0.58) | -1.13 (-1.91 to -0.35) | -1.09 (-1.67 to -0.51) | -1.09 (-1.83 to -0.33) |
| Myanmar | -2.29 (-2.7 to -1.87) | -3.51 (-4.11 to -2.91) | -2.16 (-2.56 to -1.77) | -3.44 (-4.02 to -2.85) |
| Namibia | -0.91 (-1.97 to 0.16) | -1.95 (-4.36 to 0.51) | -0.95 (-2.01 to 0.13) | -1.94 (-4.34 to 0.51) |
| Nauru | -0.02 (-0.03 to -0.01) | -0.12 (-0.14 to -0.09) | -0.01 (-0.02 to 0) | -0.12 (-0.14 to -0.1) |
| Nepal | -0.83 (-1.48 to -0.17) | -0.47 (-1.81 to 0.89) | -0.79 (-1.41 to -0.17) | -0.5 (-1.75 to 0.78) |
| Netherlands | -0.01 (-0.04 to 0.02) | 0.03 (-0.02 to 0.08) | -0.01 (-0.03 to 0.01) | 0 (-0.04 to 0.04) |
| New Zealand | 1.47 (1.22 to 1.73) | 4.31 (3.49 to 5.14) | 1.43 (1.18 to 1.68) | 4.22 (3.42 to 5.03) |
| Nicaragua | 1.47 (0.8 to 2.14) | 3.92 (1.97 to 5.91) | 1.42 (0.79 to 2.05) | 3.83 (1.94 to 5.76) |
| Niger | -1.1 (-1.62 to -0.57) | -0.42 (-1.13 to 0.3) | -1.17 (-1.71 to -0.63) | -0.46 (-1.18 to 0.26) |
| Nigeria | -0.96 (-1.29 to -0.63) | -1.14 (-1.55 to -0.73) | -0.94 (-1.26 to -0.62) | -1.12 (-1.51 to -0.72) |
| Niue | 0 (-0.02 to 0.02) | -0.15 (-0.17 to -0.14) | 0 (-0.02 to 0.01) | -0.17 (-0.18 to -0.15) |
| North Macedonia | -0.01 (-0.02 to 0.01) | 0.07 (0.05 to 0.09) | 0 (-0.02 to 0.01) | 0.07 (0.05 to 0.09) |
| Northern Mariana Islands | -0.03 (-0.04 to -0.02) | -0.05 (-0.08 to -0.02) | -0.03 (-0.04 to -0.02) | -0.05 (-0.08 to -0.02) |
| Norway | -0.06 (-0.09 to -0.03) | -0.13 (-0.27 to 0.01) | -0.04 (-0.07 to -0.01) | -0.13 (-0.26 to -0.01) |
| Oman | 0.02 (0.01 to 0.03) | -0.19 (-0.22 to -0.17) | 0.01 (0.01 to 0.02) | -0.2 (-0.22 to -0.18) |
| Pakistan | -3.46 (-6.37 to -0.46) | -5.6 (-10.05 to -0.92) | -3.3 (-6.2 to -0.32) | -5.46 (-9.92 to -0.78) |
| Palau | 0.01 (0 to 0.02) | -0.1 (-0.12 to -0.07) | 0 (-0.01 to 0.01) | -0.12 (-0.14 to -0.09) |
| Palestine | -0.02 (-0.03 to -0.01) | 0.07 (0.05 to 0.09) | -0.03 (-0.04 to -0.02) | 0.07 (0.04 to 0.09) |
| Panama | -0.06 (-0.07 to -0.06) | -0.06 (-0.09 to -0.04) | -0.06 (-0.07 to -0.05) | -0.07 (-0.1 to -0.05) |
| Papua New Guinea | -1.12 (-1.43 to -0.81) | -1.96 (-2.49 to -1.43) | -1.06 (-1.36 to -0.77) | -1.86 (-2.36 to -1.35) |
| Paraguay | 1.54 (1.12 to 1.97) | 1.95 (1.43 to 2.46) | 1.49 (1.08 to 1.9) | 1.91 (1.41 to 2.42) |
| Peru | 2.33 (1.89 to 2.77) | 7.12 (5.52 to 8.74) | 2.25 (1.85 to 2.66) | 7.08 (5.54 to 8.65) |
| Philippines | 5.33 (3.27 to 7.44) | 6.57 (3.8 to 9.41) | 5.28 (3.29 to 7.3) | 6.52 (3.82 to 9.29) |
| Poland | 0.97 (0.7 to 1.24) | 1.39 (0.98 to 1.8) | 0.86 (0.63 to 1.1) | 1.29 (0.91 to 1.67) |
| Portugal | -0.06 (-0.09 to -0.04) | -0.24 (-0.31 to -0.18) | -0.04 (-0.07 to -0.02) | -0.26 (-0.33 to -0.2) |
| Puerto Rico | 0.01 (-0.01 to 0.02) | -0.05 (-0.06 to -0.03) | 0.02 (0 to 0.03) | -0.05 (-0.06 to -0.03) |
| Qatar | 0.01 (-0.01 to 0.02) | 0.09 (0.07 to 0.12) | 0.01 (0 to 0.02) | 0.08 (0.06 to 0.11) |
| Republic of Korea | -0.18 (-0.28 to -0.08) | -0.14 (-0.33 to 0.05) | -0.15 (-0.24 to -0.06) | -0.16 (-0.35 to 0.02) |
| Republic of Moldova | 0.86 (0.14 to 1.58) | 1.43 (0.35 to 2.52) | 0.78 (0.13 to 1.43) | 1.33 (0.33 to 2.35) |
| Romania | -0.04 (-0.06 to -0.02) | 0.25 (0.14 to 0.35) | -0.02 (-0.04 to -0.01) | 0.24 (0.14 to 0.34) |
| Russian Federation | -0.02 (-0.03 to -0.01) | 0.26 (0.25 to 0.28) | -0.01 (-0.02 to 0.01) | 0.24 (0.22 to 0.26) |
| Rwanda | -1.34 (-1.85 to -0.82) | -0.61 (-1.92 to 0.72) | -1.35 (-1.87 to -0.83) | -0.71 (-2.04 to 0.63) |
| Saint Kitts and Nevis | 0.01 (-0.01 to 0.02) | 0.04 (0.01 to 0.06) | 0.01 (-0.01 to 0.02) | 0.04 (0.01 to 0.06) |
| Saint Lucia | -0.01 (-0.02 to 0.01) | 0.11 (0.09 to 0.13) | 0 (-0.02 to 0.01) | 0.1 (0.08 to 0.12) |
| Saint Vincent and the Grenadines | 0 (-0.02 to 0.01) | -0.03 (-0.05 to -0.01) | 0 (-0.01 to 0.01) | -0.03 (-0.06 to -0.01) |
| Samoa | -0.04 (-0.05 to -0.03) | -0.07 (-0.08 to -0.05) | -0.03 (-0.04 to -0.02) | -0.08 (-0.09 to -0.06) |
| San Marino | -0.06 (-0.08 to -0.03) | -0.12 (-0.18 to -0.05) | -0.05 (-0.07 to -0.03) | -0.14 (-0.19 to -0.08) |
| Sao Tome and Principe | -0.2 (-0.64 to 0.24) | -0.06 (-1.27 to 1.17) | -0.19 (-0.61 to 0.23) | -0.06 (-1.25 to 1.14) |
| Saudi Arabia | 0.01 (-0.01 to 0.02) | -0.02 (-0.04 to 0.01) | 0 (-0.01 to 0.01) | -0.03 (-0.05 to -0.01) |
| Senegal | -0.39 (-0.7 to -0.08) | 1.69 (0.89 to 2.49) | -0.29 (-0.61 to 0.04) | 1.73 (0.91 to 2.55) |
| Serbia | 0.02 (0.01 to 0.04) | -0.1 (-0.16 to -0.04) | 0.03 (0.01 to 0.04) | -0.1 (-0.16 to -0.05) |
| Seychelles | 0.04 (0.02 to 0.06) | -0.26 (-0.28 to -0.24) | 0.04 (0.02 to 0.06) | -0.26 (-0.28 to -0.23) |
| Sierra Leone | -0.86 (-1.32 to -0.4) | -0.6 (-1.22 to 0.02) | -0.85 (-1.31 to -0.39) | -0.61 (-1.22 to 0.02) |
| Singapore | -0.13 (-0.24 to -0.02) | 0.07 (-0.21 to 0.35) | -0.13 (-0.22 to -0.03) | 0.05 (-0.22 to 0.32) |
| Slovakia | -0.04 (-0.05 to -0.02) | -0.24 (-0.32 to -0.15) | -0.02 (-0.03 to -0.01) | -0.22 (-0.3 to -0.13) |
| Slovenia | 2.73 (2.21 to 3.26) | 4.81 (3.89 to 5.74) | 2.58 (2.08 to 3.07) | 4.71 (3.81 to 5.61) |
| Solomon Islands | -0.04 (-0.05 to -0.02) | 0.01 (-0.04 to 0.05) | -0.03 (-0.05 to -0.02) | 0.01 (-0.03 to 0.05) |
| Somalia | -0.04 (-0.05 to -0.03) | 0.16 (0.12 to 0.19) | -0.03 (-0.04 to -0.02) | 0.18 (0.14 to 0.21) |
| South Africa | -0.7 (-1.38 to -0.02) | -0.71 (-1.6 to 0.19) | -0.75 (-1.43 to -0.08) | -0.78 (-1.66 to 0.12) |
| South Sudan | -0.11 (-0.13 to -0.09) | 0.28 (0.24 to 0.31) | -0.09 (-0.1 to -0.07) | 0.29 (0.26 to 0.32) |
| Spain | 2.23 (1.83 to 2.63) | 4.91 (3.88 to 5.96) | 2.07 (1.7 to 2.45) | 4.73 (3.73 to 5.74) |
| Sri Lanka | 0.5 (0.21 to 0.79) | 0.46 (-0.06 to 0.99) | 0.42 (0.18 to 0.67) | 0.39 (-0.09 to 0.88) |
| Sudan | 1.67 (1.29 to 2.05) | 1.76 (1.23 to 2.29) | 1.64 (1.26 to 2.02) | 1.77 (1.25 to 2.3) |
| Suriname | -0.01 (-0.02 to 0.01) | -0.03 (-0.05 to -0.01) | 0 (-0.01 to 0.01) | -0.03 (-0.05 to -0.02) |
| Sweden | -0.03 (-0.06 to 0) | -0.03 (-0.06 to 0.01) | -0.03 (-0.05 to 0) | -0.04 (-0.07 to -0.02) |
| Switzerland | -0.07 (-0.1 to -0.03) | 0.19 (0.05 to 0.33) | -0.04 (-0.07 to -0.01) | 0.16 (0.02 to 0.29) |
| Syrian Arab Republic | 1.71 (1.33 to 2.09) | 2.55 (1.98 to 3.13) | 1.71 (1.32 to 2.1) | 2.55 (1.96 to 3.14) |
| Taiwan (Province of China) | -0.05 (-0.07 to -0.03) | -0.07 (-0.11 to -0.03) | -0.05 (-0.07 to -0.03) | -0.07 (-0.11 to -0.03) |
| Tajikistan | -1.74 (-2.02 to -1.46) | -2.8 (-3.27 to -2.32) | -1.57 (-1.83 to -1.32) | -2.63 (-3.07 to -2.18) |
| Thailand | 0.43 (0.12 to 0.75) | 0.64 (0.13 to 1.15) | 0.37 (0.11 to 0.64) | 0.53 (0.07 to 0.99) |
| Timor-Leste | -0.22 (-0.68 to 0.23) | -0.43 (-1.02 to 0.16) | -0.19 (-0.63 to 0.26) | -0.42 (-1.01 to 0.17) |
| Togo | 0.23 (-0.18 to 0.64) | 2.66 (1.74 to 3.58) | 0.29 (-0.1 to 0.69) | 2.66 (1.76 to 3.56) |
| Tokelau | -0.04 (-0.05 to -0.03) | -0.11 (-0.13 to -0.1) | -0.04 (-0.05 to -0.03) | -0.13 (-0.14 to -0.11) |
| Tonga | 0.01 (-0.01 to 0.03) | -0.1 (-0.11 to -0.08) | 0 (-0.02 to 0.02) | -0.1 (-0.12 to -0.08) |
| Trinidad and Tobago | 0.42 (0.18 to 0.67) | 0.6 (0.27 to 0.93) | 0.36 (0.15 to 0.58) | 0.55 (0.25 to 0.85) |
| Tunisia | 1.48 (1.33 to 1.64) | 2.3 (2.03 to 2.58) | 1.46 (1.28 to 1.63) | 2.25 (1.99 to 2.51) |
| Turkey | 0.31 (-0.19 to 0.82) | 0.69 (-0.36 to 1.75) | 0.11 (-0.36 to 0.59) | 0.47 (-0.52 to 1.47) |
| Turkmenistan | -0.01 (-0.02 to 0) | 0.28 (0.24 to 0.33) | -0.01 (-0.02 to 0) | 0.26 (0.22 to 0.31) |
| Tuvalu | -0.02 (-0.03 to -0.01) | -0.08 (-0.1 to -0.07) | -0.02 (-0.03 to 0) | -0.08 (-0.1 to -0.06) |
| Uganda | -3.1 (-3.35 to -2.85) | -4.93 (-5.29 to -4.57) | -3.28 (-3.54 to -3.02) | -5.02 (-5.39 to -4.65) |
| Ukraine | 1.25 (0.75 to 1.75) | 1.88 (1.27 to 2.49) | 1.19 (0.71 to 1.68) | 1.8 (1.2 to 2.4) |
| United Arab Emirates | 0.02 (0.01 to 0.03) | -0.08 (-0.1 to -0.07) | 0.01 (0 to 0.02) | -0.09 (-0.11 to -0.08) |
| United Kingdom | -0.02 (-0.06 to 0.01) | -0.08 (-0.27 to 0.1) | -0.01 (-0.05 to 0.02) | -0.09 (-0.27 to 0.09) |
| United Republic of Tanzania | -1.49 (-1.76 to -1.23) | -2.16 (-2.31 to -2.01) | -1.5 (-1.75 to -1.24) | -2.12 (-2.28 to -1.96) |
| United States of America | 0.89 (0.2 to 1.59) | 3.3 (1.74 to 4.89) | 0.82 (0.12 to 1.53) | 3.19 (1.63 to 4.78) |
| United States Virgin Islands | -0.05 (-0.06 to -0.03) | 0.14 (0.12 to 0.15) | -0.03 (-0.04 to -0.02) | 0.12 (0.1 to 0.14) |
| Uruguay | -0.06 (-0.12 to 0) | -0.15 (-0.24 to -0.05) | -0.05 (-0.11 to 0) | -0.19 (-0.28 to -0.09) |
| Uzbekistan | 0.84 (0.53 to 1.16) | 2.04 (1.35 to 2.73) | 0.72 (0.45 to 0.99) | 1.89 (1.26 to 2.53) |
| Vanuatu | 0.03 (0.01 to 0.05) | -0.15 (-0.17 to -0.13) | 0.04 (0.02 to 0.06) | -0.14 (-0.16 to -0.12) |
| Venezuela (Bolivarian Republic of) | -0.06 (-0.07 to -0.05) | 0.13 (0.08 to 0.19) | -0.05 (-0.06 to -0.04) | 0.12 (0.07 to 0.17) |
| Viet Nam | 1.55 (0.99 to 2.11) | 2.07 (1.16 to 3) | 1.48 (0.94 to 2.03) | 2.01 (1.1 to 2.92) |
| Yemen | 0.25 (-0.4 to 0.9) | 0.39 (-0.4 to 1.18) | 0.19 (-0.48 to 0.86) | 0.34 (-0.46 to 1.15) |
| Zambia | -2.48 (-3.28 to -1.67) | -3.28 (-4.57 to -1.98) | -2.53 (-3.33 to -1.73) | -3.3 (-4.58 to -2.01) |
| Zimbabwe | -0.8 (-1.29 to -0.31) | -1.51 (-2.49 to -0.52) | -0.83 (-1.31 to -0.34) | -1.48 (-2.44 to -0.5) |

ASPR, age-standardized prevalence rate; ASDR, age-standardized disability-adjusted life-years rate; CI, confidence interval; EAPC estimated annual percentage change

**Table S7** Prevalence and DALYs of infertility in five SDI regions from 1990 to 2021

| **Sex** | **Region** | **Prevalence (95% UI), 1990** | **ASPR/100,000 persons (95% UI), 1990** | **Prevalence (95% UI), 2021** | **ASPR/100,000 persons (95% UI), 2021** | **EAPC of ASPR (95% CI), 1990–2021** | **DALYs (95% UI), 1990** | **ASDR/100,000 persons (95% UI), 1990** | **DALYs (95% UI), 2021** | **ASDR/100,000 persons (95% UI), 2021** | **EAPC of ASDR (95% CI), 1990–2021** |
| --- | --- | --- | --- | --- | --- | --- | --- | --- | --- | --- | --- |
| Male | Low SDI | 2,553,100 (1,625,425 - 3,816,042) | 1,245.8 (798.69 -1,859.93) | 6,427,824 (3,885,082 - 9,979,635) | 1,269.37 (770.19 - 1,959.84) | -0.17 (-0.45 - 0.12) | 14,805 (5,551 - 33,573) | 7.13 (2.73 -16.32) | 37,248 (13,729 - 85,517) | 7.28 (2.7 - 16.88) | -0.16 (-0.44 - 0.12) |
|  | Low-middle SDI | 5,483,316 (3,304,295 - 8,526,906) | 1,019.46 (625.06 - 1,572.81) | 14,297,089 (8,370,702 - 22,875,418) | 1,395.96 (813.3 -2,252.12) | 1 (0.61 - 1.4) | 32,734 (12,419 - 74,440) | 6.01 (2.27 -13.79) | 84,065 (30,521 - 196,576) | 8.18 (2.98 - 19.2) | 0.95 (0.57 - 1.33) |
|  | Middle SDI | 10,643,923 (6,194,862 - 17,124,722) | 1,189.81 (698.93 - 1,920.87) | 18,151,666 (10,796,198 - 29,302,986) | 1,387.96 (819.52 - 2,230.9) | 0.6 (0.52 - 0.69) | 60,482 (21,611 - 143,641) | 6.7 (2.43 - 16.14) | 103,980 (38,249 - 249,703) | 7.98 (2.9 - 19.24) | 0.66 (0.58 - 0.75) |
|  | High-middle SDI | 8,274,380 (4,805,665 - 13,829,973) | 1,402.44 (813.39 - 2,341.64) | 10,194,899 (5,795,901 - 17,173,215) | 1,472.79 (851.46 - 2,414.28) | 0.13 (0.11 - 0.14) | 46,705 (16,702 - 115,887) | 7.9 (2.88 - 19.66) | 57,219 (21,039 - 141,093) | 8.35 (3.02 - 20.74) | 0.14 (0.13 - 0.16) |
|  | High SDI | 4,507,508 (2,671,002 - 7,483,432) | 913.1 (540.97 -1,503.48) | 5,890,821 (3,376,658 - 10,016,230) | 1,071.23 (617.22 - 1,766.67) | 0.63 (0.51 - 0.75) | 26,979 (10,068 - 64,671) | 5.48 (2.05 -13.12) | 34,878 (12,752 - 84,167) | 6.4 (2.3 - 15.53) | 0.6 (0.48 - 0.72) |
| Female | Low SDI | 4,687,801 (2,748,180 - 7,808,070) | 2,220.14 (1,287.09 - 3,716.05) | 12,249,744 (6,801,478 - 20,619,052) | 2,333.95 (1,299.03 - 3,969.36) | 0.1 (-0.28 - 0.5) | 25,922 (9,533 - 58,929) | 12.12 (4.52 - 27.6) | 67,696 (24,767 - 161,485) | 12.8 (4.73 - 31.1) | 0.11 (-0.28 - 0.5) |
|  | Low-middle SDI | 10,739,460 (6,073,679 - 17,862,342) | 2,046.98 (1,142.61 - 3,550.73) | 30,053,933 (16,629,265 - 51,679,485) | 2,940.52 (1,623.86 - 5,103.43) | 1.24 (0.69 - 1.79) | 60,815 (21,169 - 143,024) | 11.45 (4.01 - 27.23) | 167,400 (58,563 - 403,460) | 16.34 (5.7 - 39.57) | 1.19 (0.65 - 1.73) |
|  | Middle SDI | 22,576,313 (12,416,219 - 39,052,724) | 2,664.97 (1,427.1 - 4,649.19) | 39,038,802 (20,324,320 - 70,133,766) | 3,027.65 (1,579.65 - 5,404.99) | 0.58 (0.46 - 0.7) | 121,994 (42,876 - 302,346) | 14.3 (4.94 - 35.35) | 211,708 (75,088 - 517,044) | 16.47 (5.84 - 39.99) | 0.62 (0.5 - 0.74) |
|  | High-middle SDI | 16,546,621 (8,904,341 - 29,353,027) | 2,899.74 (1,553.44 - 5,158.24) | 21,200,266 (10,465,956 - 38,450,758) | 3,180.18 (1,643.03 - 5,750.65) | 0.26 (0.24 - 0.28) | 88,611 (30,730 - 229,796) | 15.51 (5.41 - 40.19) | 112,846 (38,219 - 282,874) | 17.04 (5.87 - 41.71) | 0.27 (0.24 - 0.29) |
|  | High SDI | 5,090,008 (2,095,085 - 10,270,333) | 1,049.61 (433.47 - 2,114.52) | 7,476,943 (2,888,374 - 14,892,481) | 1,415.8 (542.85 - 2,854.67) | 1.43 (1.28 - 1.58) | 28,320 (8,576 - 75,480) | 5.85 (1.77 - 15.46) | 41,101 (11,612 - 110,625) | 7.83 (2.2 - 21.41) | 1.41 (1.25 - 1.56) |

DALYs, disability-adjusted life-years; ASPR, age-standardized prevalence rate; ASDR, age-standardized disability-adjusted life-years rate; UI, uncertainty interval; CI, confidence interval; EAPC estimated annual percentage change


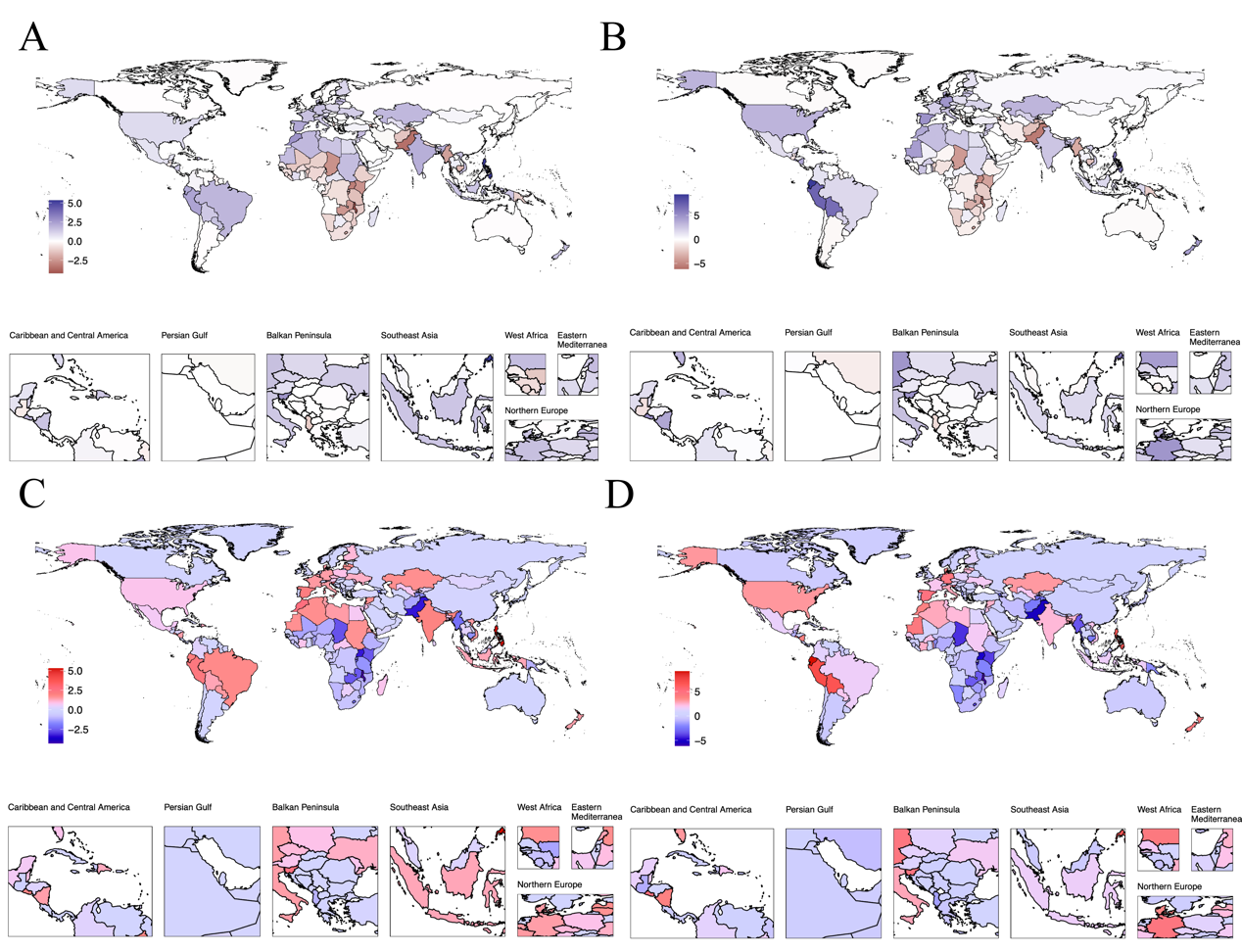


**Fig. S1** Estimated annual percentage changes of age-standardised prevalence and disability-adjusted life-years rates from 1990 to 2021 for male and female infertility. Estimated annual percentage changes of age-standardised prevalence for male infertility (A) and female infertility (B); age-standardised rates of disability-adjusted life-years for male infertility (C) and female infertility (D).


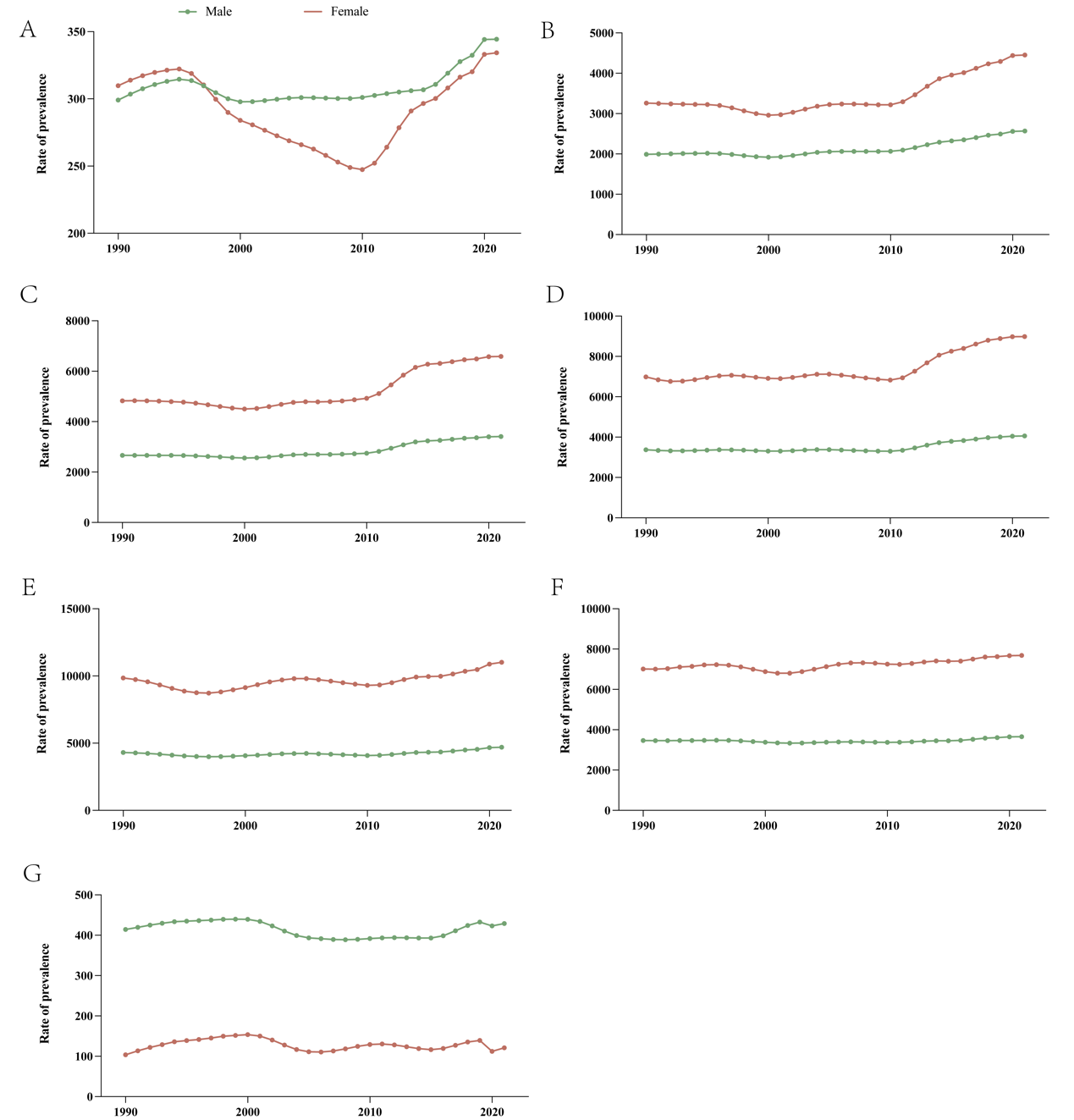


**Fig. S2** Time trend of prevalence rates in each age subgroup from 1990 to 2021. 15-19 years (A), 20-24 years (B), 25-29 years (C), 30-34 years (D), 35-39 years (E), 40-44 years (F), and 45-49 years (G).


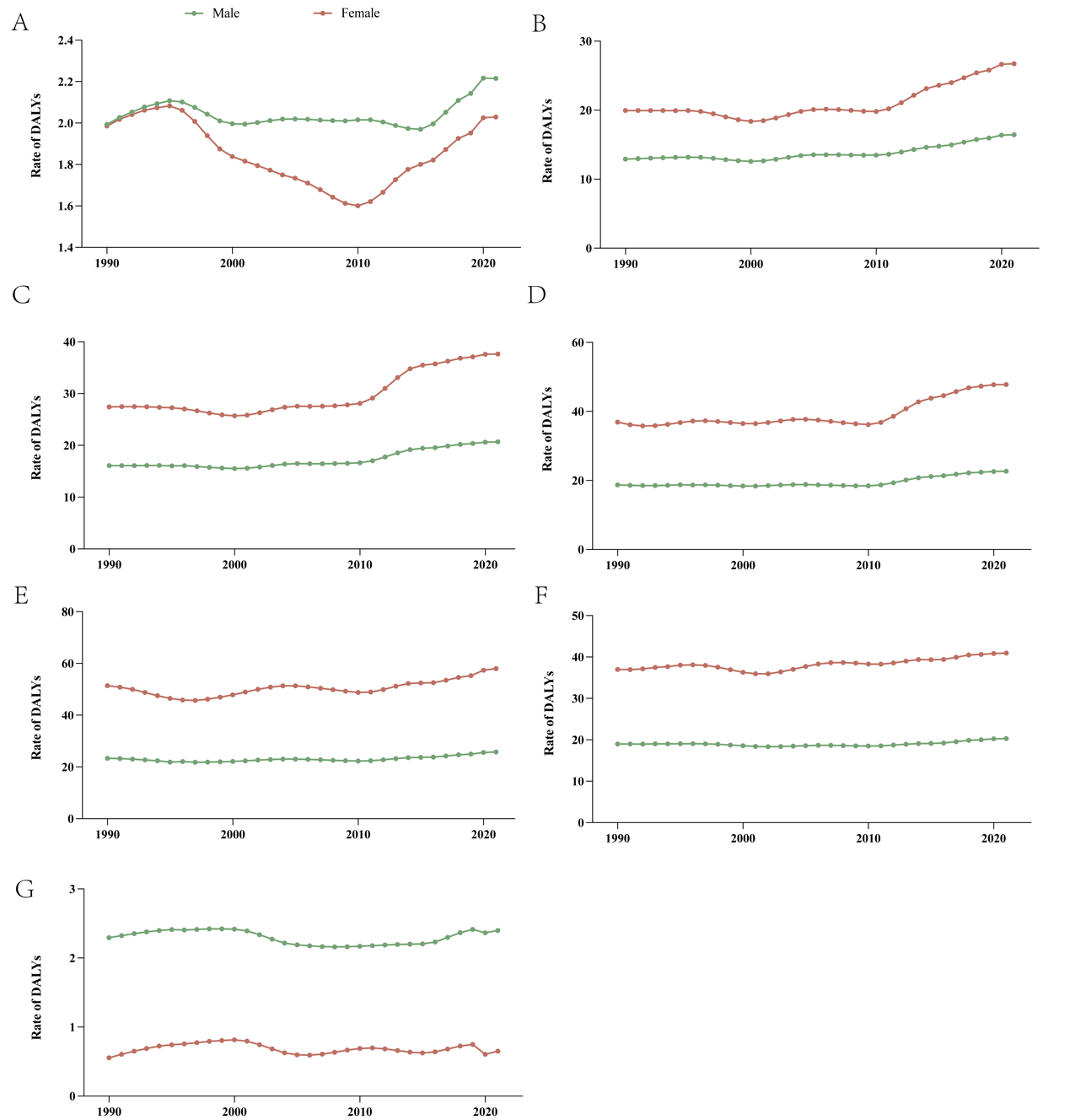


**Fig. S3** Time trend of DALYs rates in each age subgroup from 1990 to 2021. 15-19 years (A), 20-24 years (B), 25-29 years (C), 30-34 years (D), 35-39 years (E), 40-44 years (F), and 45-49 years (G). DALYs, disability-adjusted life-years


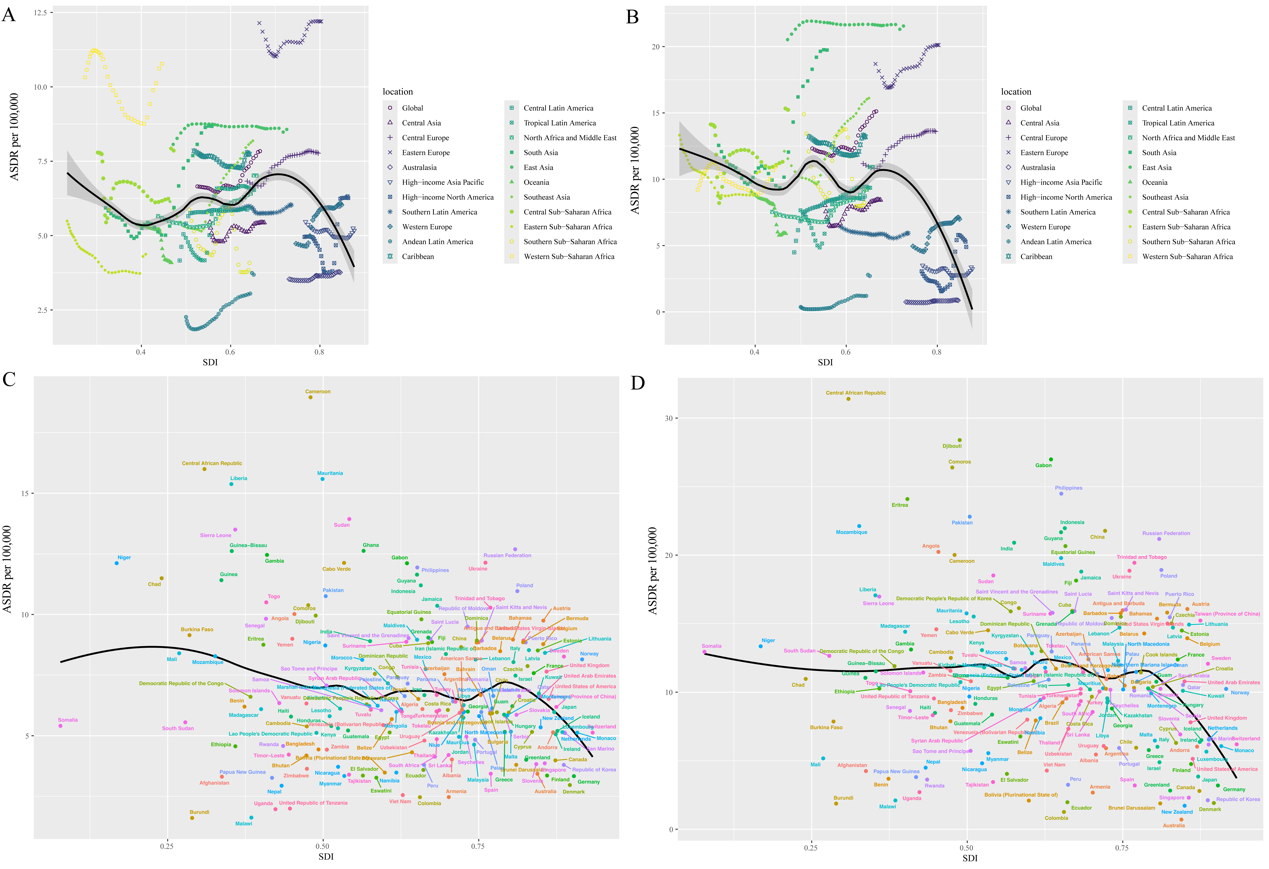


**Fig. S4** ASDRs for male and female fertility of 21 regions and 204 countries and territories by SDI. ASDRs for male (A) and female (B) fertility of 21 regions from 1990−2021 by SDI; ASDRs for male (C) and female (D) fertility of 204 countries and territories in 2021 according to the SDI. ASDR, age-standardized disability-adjusted life-years rate; SDI, Socio-demographic index
